# Chalcogenides by Reduction of their Dioxides in Ultra‐Alkaline Media

**DOI:** 10.1002/anie.202107642

**Published:** 2021-09-06

**Authors:** Ralf Albrecht, Michael Ruck

**Affiliations:** ^1^ Faculty of Chemistry and Food Chemistry Technische Universität Dresden 01069 Dresden Germany; ^2^ Max-Planck Institute for Chemical Physics of Solids Nöthnitzer Straße 40 01187 Dresden Germany

**Keywords:** crystal structure, hydroflux, selenium, spectroscopy, tellurium

## Abstract

The reaction of chalcogen dioxides *Ch*O_2_ (*Ch*=Se, Te) with As_2_O_3_ in a 30 molar KOH hydroflux at about 200 °C yielded crystals of potassium trichalcogenides K_2_ *Ch*_3_ with dimensions up to 2 cm. Arsenic trioxide acts as electron donor and is oxidized to arsenate(V). The new heterochalcogenide anion (TeSe_2_)^2−^ formed when starting from SeO_2_ and TeO_2_ simultaneously. The compound K_2_TeSe_2_ crystallizes isostructural to K_2_S_3_ and K_2_Se_3_. The unexpected redox reaction as well as the precipitation of hygroscopic compounds from an aqueous solution are attributed to a strongly reduced activity of water. The reactions were studied by Raman and UV/Vis spectroscopy. Depending on the concentration of As_2_O_3_, colorless monochalcogenide *Ch* ^2−^ or orange Se_2_^2−^ and purple Te_2_^2−^ anions are dominating the solutions.

## Graphical abstract

The unexpected reduction of selenium(IV) and tellurium(IV) oxides to chalcogenides was achieved under ultra‐alkaline conditions by using As_2_O_3_ as reducing agent. By optimization of the reaction conditions, large crystals of K_2_Se_3_, K_2_Te_3_ and K_2_Se_2_Te were grown within two days. In the latter compound, the novel mixed chalcogenide anion (SeTe)^2−^ was discovered.

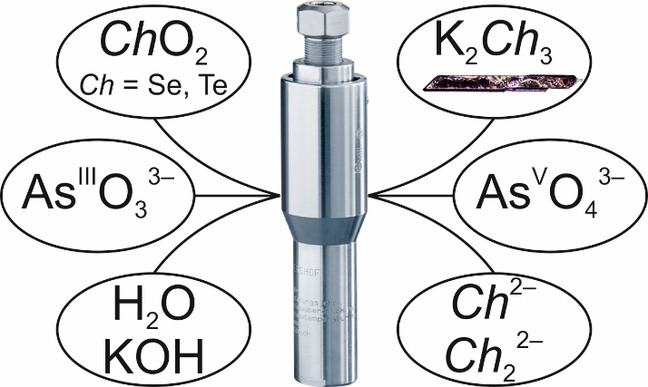

## Introduction

The history of alkali metal selenides and tellurides reaches back at least to the beginning of the last century, when the groups of *Zintl* and *Klemm* obtained such compounds from reactions in liquid ammonia at −78 °C.[^1^, ^2^, ^3^] They were able to isolate single‐crystals and to determine the crystal structures of several alkali metal chalcogenides *A*_2_ *Ch* (*A*=Li—K, *Ch*=Se, Te), which contain closed‐shell *Ch* ^2−^ anions. Forty years later, single‐crystals of the triselenide K_2_Se_3_ were synthesized under ammonothermal conditions (150 °C, 500 bar) in an autoclave starting from the elements.^[4]^ Concurrently, the first tritelluride K_2_Te_3_ was obtained from the elements at about 600 °C in a welded iron autoclave.^[5]^ The trichalcogenide anions *Ch_3_* ^2−^ are angulated molecules (Se_3_^2−^ 102.5°; Te_3_^2−^ 104.4°)[^4^, ^5^] with a negative (formal) charge on each of the terminal atoms. Using similar procedures, single‐crystals of several oligoselenides and tellurides were synthesized, for example, K_2_ *Ch*_2_,^[6]^ K_5_ *Ch*_3_ (*Ch*=Se, Te)[^7^, ^8^] and also the hetero‐trichalcogenide K_2_TeSe_3_.^[9]^ Alternatively, potassium chalcogenides, such as β‐K_2_Se_2_ or K_2_Se_4_, were obtained by solvothermal synthesis in an organic solvent, for example, *N*,*N*‐dimethylformamide (DMF) or ethane‐1,2‐diamine.^[10]^ As the starting materials and reaction products are sensitive to oxygen and moisture, all described methods necessitate consequent handling under inert gas to exclude water.

Accordingly, we were highly surprised to obtain such sensitive compounds with reduced chalcogen species from an aqueous medium starting from chalcogen(IV) oxides. We had applied the new hydroflux method,^[11]^ which utilizes a highly concentrated mixture of alkali metal hydroxide and water with a molar ratio *q*(*A*)=*n*(H_2_O):*n*(*A*OH) close to one (i.e. 30 to 50 molar) as reaction medium. Commonly, sodium or potassium hydroxide are employed. The hydroflux provides simple and fast synthesis of crystalline metal oxides and hydroxides in almost quantitative yield.[^12^, ^13^] Typically, the hydroflux reaction is completed within 10 hours at about 200 °C in a stainless steel autoclave with a PTFE inlet, which endures the ultra‐alkaline conditions and prevents the loss of water. As the activity of the water is dramatically reduced in such aqueous salt melts, the pressure evolving during the hydroflux reaction is much lower than under hydrothermal conditions. The hydroflux medium tends to promote higher oxidations states than expected for a diluted aqueous system. For example, the oxidation of arsenic(III) and chromium(III) to their maximum oxidation states was observed.[^14^, ^15^]

In the following, we report studies on the reduction of SeO_2_ and TeO_2_ by As_2_O_3_ in a KOH hydroflux, resulting in crystals of K_2_Se_3_, K_2_Te_3_ and the new hetero‐trichalcogenide K_2_TeSe_2_.

## Results and Discussion

To understand the reactions in the hydroflux systems, a brief review of the chemistry of chalcogens under reductive conditions in various other media is helpful.

In liquid ammonia, solutions of tellurides adapt a characteristic color depending on the anionic species formed. When adding an alkali metal *A* to liquid ammonia, an intense blue color caused by solvated electrons appears immediately. The latter are able to reduce tellurium to form different tellurides: Te^2−^ anions form a white precipitate (*A*_2_Te), Te_2_^2−^ anions turn the solution deep violet‐blue, and Te_3_^2−^ anions have an intense wine‐red color.^[16]^ When DMF is used as solvent, no sequence of differently colored solutions occurs, as firstly the starting elements are insoluble in DMF and secondly no solvated electrons are present in the solution. When stirring elemental tellurium and an alkali metal in a DMF solution at room temperature, in the beginning no reaction is observed visually.^[17]^ After half an hour, the DMF solution develops a faint pink color that intensifies to deep purple over the course of hours. The tellurium and alkali metal appear to react by physical contact resulting in the formation of Te^2−^ anions, which are soluble in DMF.^[18]^ Those monotelluride anions react with remaining tellurium metal to form oligotellurides, which are linked by the equilibrium 1:

(1) $$\mathrm{Te}{}^{2-}\;+\;\mathrm{Te}{}_{3}{}^{2-}\;\vec{\leftarrow }\;2\;\mathrm{Te}{}_{2}{}^{2-}$$

When pre‐synthesized alkali metal tellurides are dissolved in DMF, the same deep purple colored solutions form, independent of the *A*:Te ratio and the nature of the alkali metal *A*.^[18]^

In contrast to the tellurides, the chemistry of selenides in O_2_‐free aqueous systems was investigated in several spectroscopic and electrochemical studies.[^19^, ^20^, ^21^, ^22^, ^23^] In those experiments, dissolved H_2_Se was oxidized by H_2_O_2_ or reacted with elemental selenium. The equilibria 2 and 3 between four species Se_n_^2−^ with *n=*1–4 in low‐concentrated alkaline solutions were proposed:^[20]^

(2) $$\mathrm{Se}{}^{2-}\;+\;\mathrm{Se}{}_{3}{}^{2-}\;\vec{\leftarrow }\;2\;\mathrm{Se}{}_{2}{}^{2-}$$

(3) $$\mathrm{Se}{}^{2-}\;+\;2\;\mathrm{Se}{}_{4}{}^{2-}\;\vec{\leftarrow }\;3\;\mathrm{Se}{}_{3}{}^{2-}$$

At room temperature and at pH 14, the product sides of both equilibria are preferred.[^19^, ^20^] In the range between pH 7 and pH 5, an approximately equal concentration of diselenide Se_2_^2−^ and triselenide Se_3_^2−^ anions was observed.^[21]^ The protonated selenides species HSe^−^ (p*K*_a_=15.0) and HSe_2_^−^ (p*K*_a_=9.3) were observed even in 1 m KOH solutions, however, when doubling the base concentration both anions were essentially deprotonated.[^19^, ^23^]

In our experiments, the syntheses of selenides and tellurides were performed under hydroflux conditions in a stainless‐steel autoclave with PTFE inlet. The reaction medium consisted of a potassium hydroxide hydroflux with *q*(K)=*n*(H_2_O):*n*(KOH)=1.9 (i.e. about 30 mol L^−1^). At room temperature this is a clear solution with a small residuum of solid KOH. The starting materials SeO_2_, TeO_2_, and As_2_O_3_ are well‐soluble in highly alkaline media. The use of other reducing agents, e.g., V_2_O_3_, VO_2_, or Sb_2_O_3_, is also possible, but they differ in their solubility under hydroflux conditions. For example, V_2_O_3_ has a rather poor solubility, while its oxidation product VO_4_^3−^ is readily soluble in highly alkaline solutions. For Sb_2_O_3_, it is vice versa. As_2_O_3_ as well as As_2_O_5_ are well‐soluble in the hydroflux medium. Therefore, As_2_O_3_ was mainly applied as reducing agent. The observed solubility of these oxides under hydroflux conditions is comparable to the one in diluted alkaline solutions.[^24^, ^25^, ^26^]

**Figure 1:**
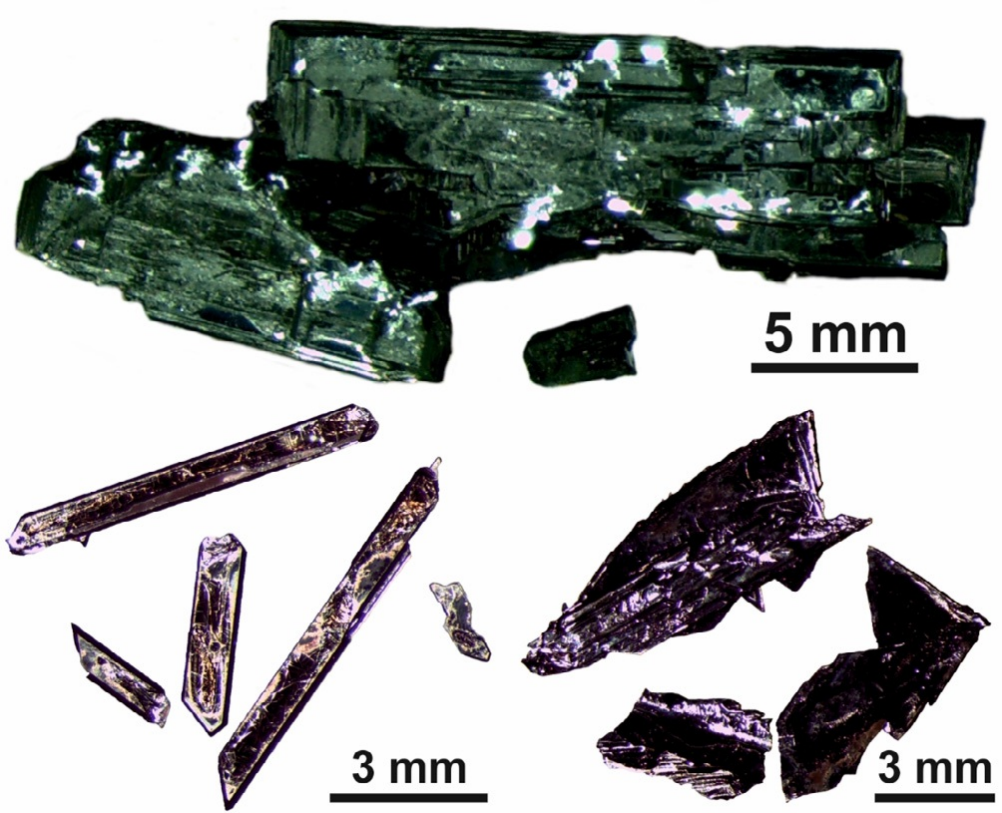
Selected crystals of K_2_Te_3_ (top), K_2_Se_3_ (bottom left) and K_2_Se_2_Te (bottom right) obtained from hydroflux syntheses.

For the synthesis of K_2_Te_3_, the molar ratio *q*(Te)= *n*(As_2_O_3_):*n*(TeO_2_)=1.2 was used. After sealing the autoclave, the mixture was reacted for 48 hours at 200 °C, before it was cooled down to room temperature within 24 h. The reaction product consisted of large black bar‐shaped crystals of K_2_Te_3_ (Figure 1) and a pale purple solution. Experiments with *q*(Te) of 1.0 or 0.75 also resulted in the crystallization of K_2_Te_3_. Despite the sub‐stoichiometric content of the reducing agent in these experiments (for an equation see below), there was no evidence of the formation of elemental tellurium. In experiments with *q*(Te) ratios larger than 1.2, K_2_Te_3_ did not form.

Substantially shorter reaction times decreased the yield of K_2_Te_3_ while the purple color of the solution intensified. The purple solutions are sensitive against water and air, resulting in the precipitation of elemental tellurium. Spectroscopic analysis of this purple solution point towards the ditelluride ion Te_2_^2−^ (see below).

The syntheses of selenides from SeO_2_ under hydroflux conditions followed the same procedure. To synthesize K_2_Se_3_, the molar ratio *q*(Se)=*n*(As_2_O_3_):*n*(SeO_2_)=1.2 was used with a reactant concentration of about *c*(SeO_2_)=1 mol L^−1^. Hence, *c*(SeO_2_) was about ten times higher than *c*(TeO_2_) needed for the crystallization of K_2_Te_3_. The reaction product consisted of a deep red solution and large K_2_Se_3_ crystals (Figure 1). Experiments with lower reactant concentrations yielded only in a deep red solution without any solid product. The red solution contained diselenide anions Se_2_^2−^ (see below).

By using a larger excess of reducing agent, the intense color of the dichalcogenide solutions vanished at *q*(*Ch*)=3.0. Furthermore, when dissolved As_2_O_3_ is added to the colored dichalcogenide solutions at room temperature, colorless solutions are obtained. Both observations indicate the formation of monochalcogenide anions *Ch* ^2−^. Equation 4 summarizes the reduction of the dichalcogenide anions:

(4) $$Ch{}_{2}{}^{2-}\;+\;\mathrm{AsO}{}_{3}{}^{3-}\;+\;2\;\mathrm{OH}{}^{-}\;\to \;2\;Ch{}^{2-}\;+\;\mathrm{AsO}{}_{4}{}^{3-}\;+\;H{}_{2}O$$

A mixture containing TeO_2_ and SeO_2_ yielded neither a mixture of K_2_Se_3_ and K_2_Te_3_ nor a solid solution with the two anions in one solid, but K_2_TeSe_2_. The small difference in the electronegativity (Pauling: Se 2.5, Te 2.1) is sufficient to assign the two elements their roles according to the charge distribution in the heteroatomic *Ch*_3_^2−^ anion. The increased intramolecular polarity in the diselenotellurate(II) ^−^(Se^−II^−Te^II^−Se^−II^)^−^ compared to the triselenide ^−^(Se^−I^−Se^0^−Se^−I^)^−^ is symbolized, but certainly also overemphasized, by the oxidation states.

K_2_TeSe_2_ was synthesized under reaction conditions similar to those of the homoatomic trichalcogenides, using SeO_2_ and TeO_2_ in the molar ratio of 2:1. Adding an excess of about 5 % of SeO_2_ helped to avoid the co‐crystallization of K_2_Te_3_, which is less soluble in the hydroflux than K_2_Se_3_. An amount of 1.3 equivalents of the reducing agent As_2_O_3_ was added (based on ^2^/_3_ SeO_2_ + ^1^/_3_ TeO_2_). Similar to the synthesis of K_2_Se_3_, relatively high reactant concentrations are necessary to obtain crystals of K_2_TeSe_2_.

It was surprising to obtain hygroscopic trichalcogenides from their dioxides in a hydroflux, which (a) contains water and (b) is usually stabilizing high oxidation states. In this case, the As_2_O_3_, which had initially been added for other reasons, acted as the reducing agent and was itself oxidized to arsenic(V). Arsenic is not only the electron donor but binds the oxygen atoms provided by the chalcogen(IV) oxides in AsO_4_^3−^ anions. This redox chemistry is far from what the standard potentials let expect, but can be rationalized by Equation 5:

(5) $$7\;\mathrm{As}{}_{2}O{}_{3}\;+\;6\;ChO{}_{2}\;+\;4\;K{}^{+}\;+\;46\;\mathrm{OH}{}^{-}\;\to 2\;K{}_{2}Ch{}_{3}\;+\;14\;\mathrm{AsO}{}_{4}{}^{3-}\;+\;23\;H{}_{2}O$$

The redox reaction is promoted by the high concentration of hydroxide ions on the side of the reactants. Moreover, the hydroflux is highly hygroscopic. The initially contained water but also water formed through the reaction are strongly bonded to hydroxide ions. Thereby, the activity of the water is considerably reduced, which does not only decrease its vapor pressure and drives the reaction but obviously prevents the hydrolysis of the water sensitive trichalcogenides. On the other hand, the reaction diluted the hydroflux so that it did not solidify upon cooling to room temperature. Washing of the reaction product with a protic solvent, for example, an alcohol, strongly increases the activity of water and thereby induces the decomposition of K_2_ *Ch*_3_. Similar observations had been made for other water sensitive products from hydroflux syntheses, for example, K_2_[Fe_2_O_3_(OH)_2_] or Tl_3_IO.[^27^, ^28^] Also several aprotic polar solvents, for example, DMF, proved to be unsuitable for rinsing because potassium hydroxide is less soluble in them than the trichalcogenides. Therefore, the products were filtered under inert conditions by using a Schlenk‐frit. The yields with respect to the used *Ch*O_2_ were 90 % for K_2_Te_3_, 60 % for K_2_Se_3_ and 80 % for K_2_Se_2_Te, related to the solubility of the diverse chalcogenide species. The adhering KOH together with the genuine moisture‐sensitivity of K_2_ *Ch*_3_ necessitated storage and handling of the crystals under inert conditions (argon). The powder X‐ray diffraction patterns of the isolated crystals showed single‐phase products, but small residuals of the hydroflux were visible in the scanning electron microscope (Figure S1 to S4, Table S1, Supporting Information).

X‐ray diffraction on black single‐crystals of K_2_Se_3_ (*Cmc*2_1_) and K_2_Te_3_ (*Pnma*) confirmed the known structures.[^4^, ^5^] For the new compound K_2_TeSe_2_ an orthorhombic structure in the non‐centrosymmetric space group *Cmc*2_1_ (no. 36) was found with the lattice parameter *a*=783.42(4) pm, *b*=1045.64(6) pm, and *c*=777.13(4) pm at 100(1) K. Details on the structure determinations and the atomic parameters of the three compounds can be found in Tables S2 to S8, Supporting Information. Selected bond lengths and angles are listed in Table S9, Supporting Information.

**Figure 2:**
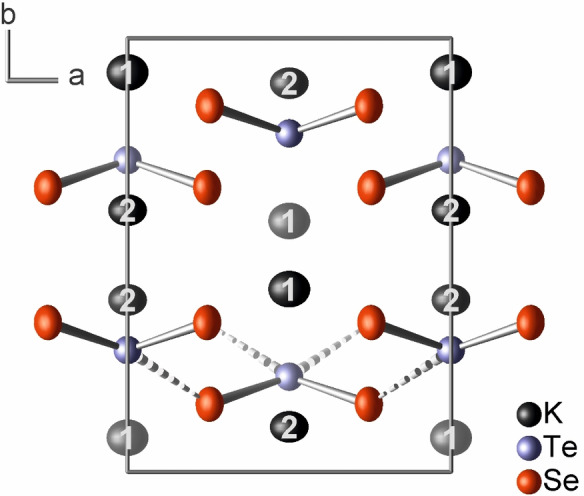
Crystal structure of K_2_TeSe_2_ projected along the [001] direction. The secondary bonds Te^II^⋅⋅⋅Se^−II^ are dotted. Ellipsoids enclose 99.99 % of the probability density of the atoms at 100(1) K.^[58]^

K_2_TeSe_2_ is isostructural to K_2_Se_3_ and K_2_S_3_ (Figure 2). The angulate diselenotellurate(II) anion, (TeSe_2_)^2−^, has crystallographic *C*_2*v*_ symmetry with two equal Se−Te bond lengths of 256.2(1) pm and a Se−Te−Se angle of 97.6(1)°. The (TeSe_2_)^2−^ anion had previously been found in (2,2,2‐crypt‐K)_2_(TeSe_2_)⋅en (en=ethylendiamin)^[29]^ and [Mn(en)_3_](TeSe_2_)^[30]^ with Te−Se bond lengths of about 250 pm and 250.3(1) pm as well as Se−Te−Se angles of 111.3(1)° and 102.6(1)°, respectively. In these structures, the (TeSe_2_)^2−^ anions are well separated from each other and interact with hydrogen atoms of the organic ligands. The wider Se−Te−Se angles are consistent with the shorter Te−Se bond lengths, which increase the repulsion between the terminal atoms. In K_2_TeSe_2_, short intermolecular distances of 333.4(1) pm suggest secondary bonds Te^II^⋅⋅⋅Se^−II^, which, together with stronger cation‐anion interactions, might be responsible for the elongated primary Te−Se bond. In alkali metal trichalcogenides *A*_2_ *Ch*_3_ (*A*=K–Cs; *Ch*=S–Te) with homonuclear anions, which have a lower intramolecular polarity than (TeSe_2_)^2−^, the shortest intermolecular distances range from 344 pm to 386 pm.^[31]^

Besides K_2_TeSe_2_, the following compounds crystallize in the K_2_S_3_ structure type: *A*_2_ *Ch*_3_ (*A*=K–Cs; *Ch*=S, Se), Cs_2_Te_3_ and Cs_2_TeS_2_.[^4^, ^31^, ^32^, ^33^] The bond angle in the *Ch*_3_^2−^ anions deceases from the sulfides (average angle of 106.0°) via the selenides (average angle of 103.1°) to the telluride (100.1° in Cs_2_Te_3_).^[31]^ This can be attributed to a decreasing s‐orbital contribution to the bonding when proceeding to the heavier main‐group elements. Despite its smaller terminal atoms, the anion in Cs_2_TeS_2_ has a slightly wider bond angle (99.4°) than (TeSe_2_)^2−^ in K_2_TeSe_2_, which might be an effect of the higher electronegativity and thus the higher partial charge of the sulfur atoms compared to selenium.

**Figure 3:**
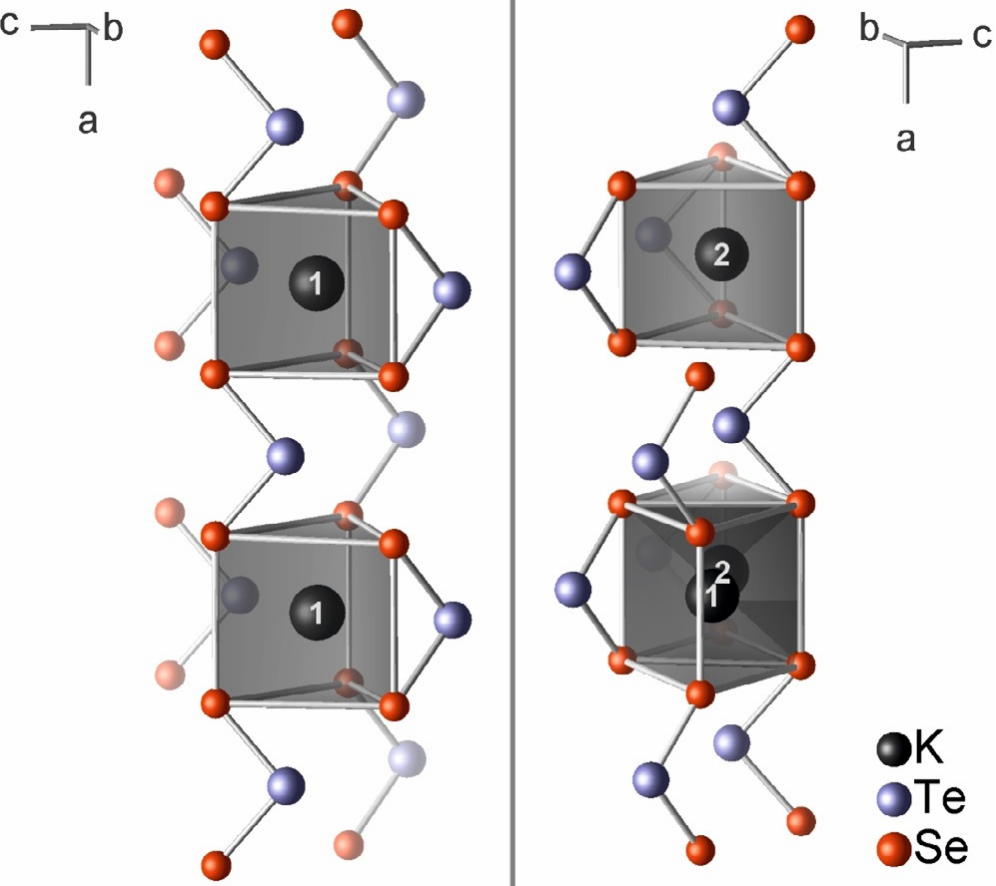
Two views of the coordination polyhedra of the potassium cations in K_2_TeSe_2_. The two potassium atoms share the uncapped square face of their trigonal prism.

In the crystal structure of K_2_TeSe_2_, the (TeSe_2_)^2−^ anions form double‐layers parallel to (010) (Figure 2) with the tellurium atoms pointing towards the inside of the double layer. The potassium atoms separate the double layers. The polarity of the structure is evident, as all (TeSe_2_)^2−^ “arrowheads” point into the same direction along [001] (Figure S5, Supporting Information). The two potassium atoms K1 and K2 are each coordinated by six selenium atoms in the shape of distorted trigonal prisms with *C*_s_ symmetry (Figure 3). Within those polyhedra, the K−Se bond lengths range from 338.9(1) pm to 360.9(1) pm and from 338.3(1) pm to 349.6(1) pm, respectively (Table S8, Supporting Information). The slightly larger polyhedron around K1 involves six (TeSe_2_)^2−^ anions, whereas only five anions form the trigonal prism around K2. Comparing the [KSe_6_] polyhedra of K_2_Se_3_ and K_2_TeSe_2_, the sum of their volumes is about 5 % larger for the latter. The [KSe_6_] prisms of K1 and K2 share the square face that is not capped by a tellurium atom (K⋅⋅⋅Te 340.8(1) to 390.7(1) pm). The [K_2_Se_8_] double prisms share corners and edges to form a three‐dimensional framework. In K_2_TeSe_2_, the shortest K⋅⋅⋅K distance is with 366.7(1) pm even shorter than in K_2_Se_3_ [369.4(1) pm], but not as short as in K_2_S_3_ [359.2(2) pm].^[4]^ In K_2_Te_3_, which crystallizes in its own structure type, the shortest distance between cations is 441.0(1) pm.

To obtain further insight into the chemical processes in the hydroflux, especially the prevalent chalcogenide anions *Ch*_n_^2−^ (*n=*1, 2, 3), the reacted solutions were analyzed by UV/Vis and Raman spectroscopy. The reaction conditions were *q*(K)=1.9 and 200 °C, as for the above syntheses, but the reaction time was only five hours. The reactant concentration of the syntheses for the UV/Vis measurements was about 0.01 mol L^−1^, while the concentration for the Raman measurements was about 30 times higher. Analyses were performed under ambient conditions in air and at room temperature. During the UV/Vis measurements, the lower limit in wavelength was about 240 nm because of strong absorption by the hydroflux medium. Raman and UV‐vis spectra of each single reactant dissolved in a hydroflux with *q*(K)=1.9 can be found in Figure S6 to S11, Supporting Information.

**Figure 4:**
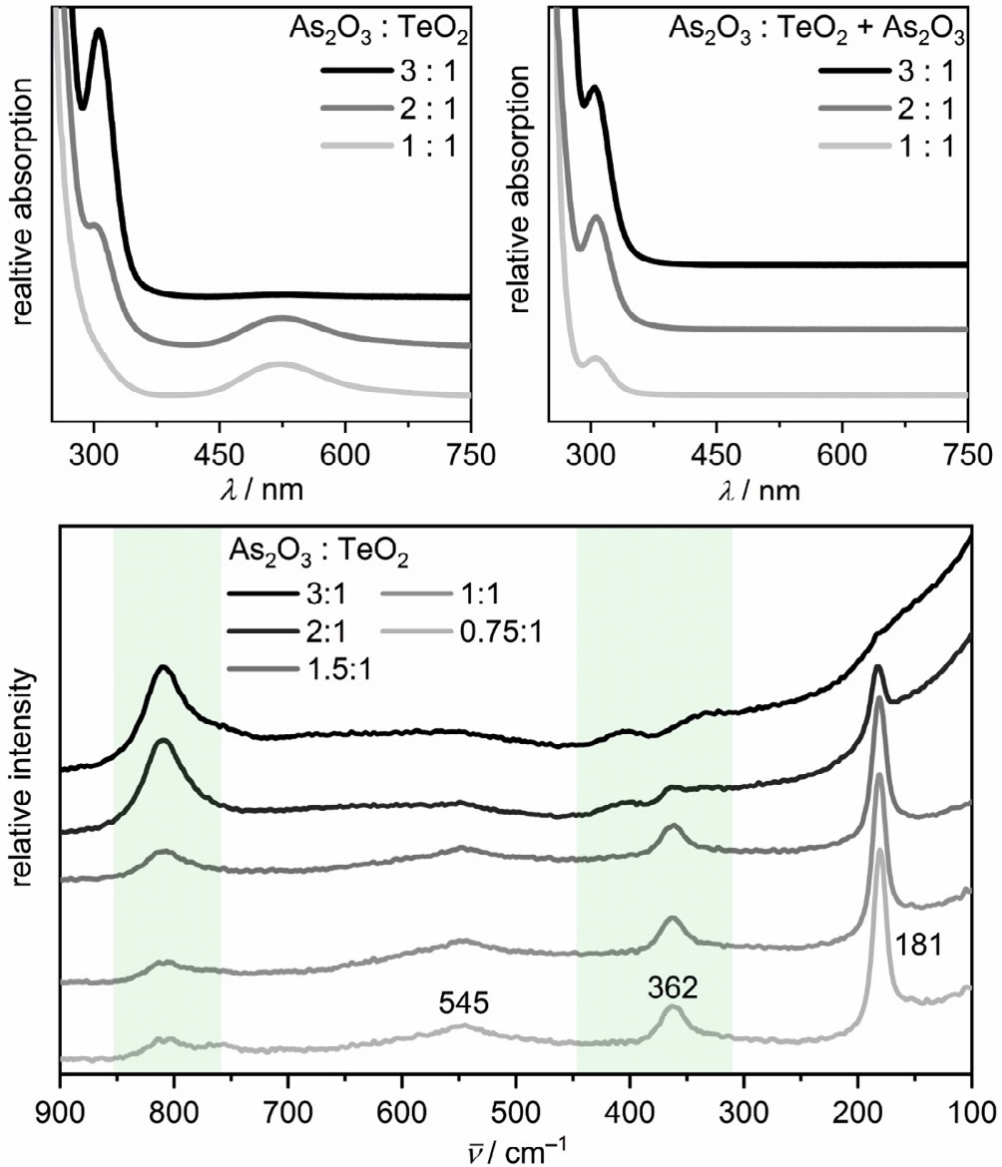
UV/Vis (top left) and Raman (bottom) spectra of telluride solutions synthesized with different *q*(Te) ratios. Absorption spectra of telluride solutions after adding dissolved As_2_O_3_ at room temperature are presented (top right). The green boxes mark the wavenumber range, where As−O vibrations of the AsO_4_^3−^ anion occur.

Purple solutions were obtained starting from a molar ratio of As_2_O_3_ and TeO_2_ of *q*(Te)=1. In this ratio, the complete oxidation of arsenic(III) to arsenic(V) provides four electrons per tellurium(IV) atom. The UV/Vis spectrum of such a sample show an absorption band with a maximum at 522 nm (Figure 4), which is slightly shifted to lower frequencies in comparison with the published values for ditelluride anions Te_2_^2−^ of 508 to 512 nm.[^34^, ^35^, ^36^, ^37^] In diluted alkaline solutions, the presence of the purple Te_2_^2−^ had been observed and analyzed in various experimental setups, e.g., as a product of accidental oxidation of monotelluride solutions by oxygen from leakage,[^34^, ^35^] after the oxidation of monotelluride solution in a photochemical cell by irradiation of CdTe,^[35]^ in an electrolysis starting from a monotelluride solution^[35]^ or during an electrolysis generating monotelluride that further reacted with elemental tellurium to form the ditelluride anion.[^36^, ^37^] Moreover, the absorption spectrum of the isoelectronic iodine molecule I_2_ dissolved in hexane exhibits a similar band with a maximum at about 520 nm.[^34^, ^38^]

The Raman spectra of experiments with *q*(Te)=0.75, 1, or 1.5 (i.e. 3, 4, 6 electrons per Te^IV^) show a vibration band at 181 cm^−1^ (Figure 4), which occurs in an energy range typical for oligotellurides.[^17^, ^39^, ^40^, ^41^] We found no literature data for Te−Te vibrations of tellurides in aqueous solutions. K_2_Te_2_ dissolved in DMF shows a vibration at 164 cm^−1^.^[17]^ The Raman vibrations of the pentatelluride ion Te_5_^2−^ in acetone are located at 170 cm^−1^ and 195 cm^−1^.^[39]^ The Te−Te bands in As−Te and Se−Te glasses were reported to occur at 155 cm^−1^ and 175 cm^−1^.[^40^, ^41^] Consequently, and as indicated by the UV/Vis spectra, we assign the band at 181 cm^−1^ to the vibration of the Te_2_^2−^ anion. The bands observed at 362 cm^−1^ and 545 cm^−1^ represent the first and second overtone of the 181 cm^−1^ vibration band, respectively. The presence of these overtones and the high intensity of the Te−Te vibration band compared to the spectra of the selenides are caused by Raman resonance of the 532 nm laser with the absorption band at 522 nm.

When the amount of reducing agent is increased, the purple color of the solution fades until a colorless solution is obtained in syntheses starting from *q*(Te)=3 (i.e. 12 electrons per Te^IV^). These solutions as well as samples for which dissolved As_2_O_3_ was added at room temperature to purple Te_2_^2−^ solutions showed a single symmetrical absorption band in the UV/Vis with a maximum at 324 nm (Figure 4). This is in good agreement with the published value of 325 nm for Te^2−^ in diluted alkaline solutions.[^34^, ^36^] In the corresponding Raman spectra, no band was detectable in the typical energy range of Te−Te vibrations, as can be expected for the monotelluride anion Te^2−^ being the predominant species. The protonated monotelluride HTe^−^ (270 nm)^[34]^ was not observed in our experiments. This can be expected for ultra‐alkaline media, as the second acid dissociation constant p*K*_a2_ of hydrogen telluride H_2_Te had been reported to be 12.2.^[42]^

In none of the solutions, the tritelluride anion Te_3_^2−^ could be detected spectroscopically. It had been reported to have an UV/Vis absorption band at 376 nm in DMF,^[18]^ and K_2_Te_3_ dissolved in liquid ammonia or DMF exhibits a vibration band at about 162 cm^−1^.^[17]^ In the hydroflux medium, the slow crystallization of K_2_Te_3_ during the synthesis at 200 °C and its insolubility at room temperature suggest an equilibrium between the telluride species similar to Equation (1), in which the tritelluride anion is non‐preferential. The precipitation of K_2_Te_3_ is thus not caused by a high concentration of Te_3_^2−^ but a very small solubility product. These observation are in line with reported electrochemical experiments on the reductive dissolution of a tellurium cathode: while at pH 9 mainly Te^2−^ anions formed, Te_2_^2−^ anions dominated above pH 12.^[43]^

**Figure 5:**
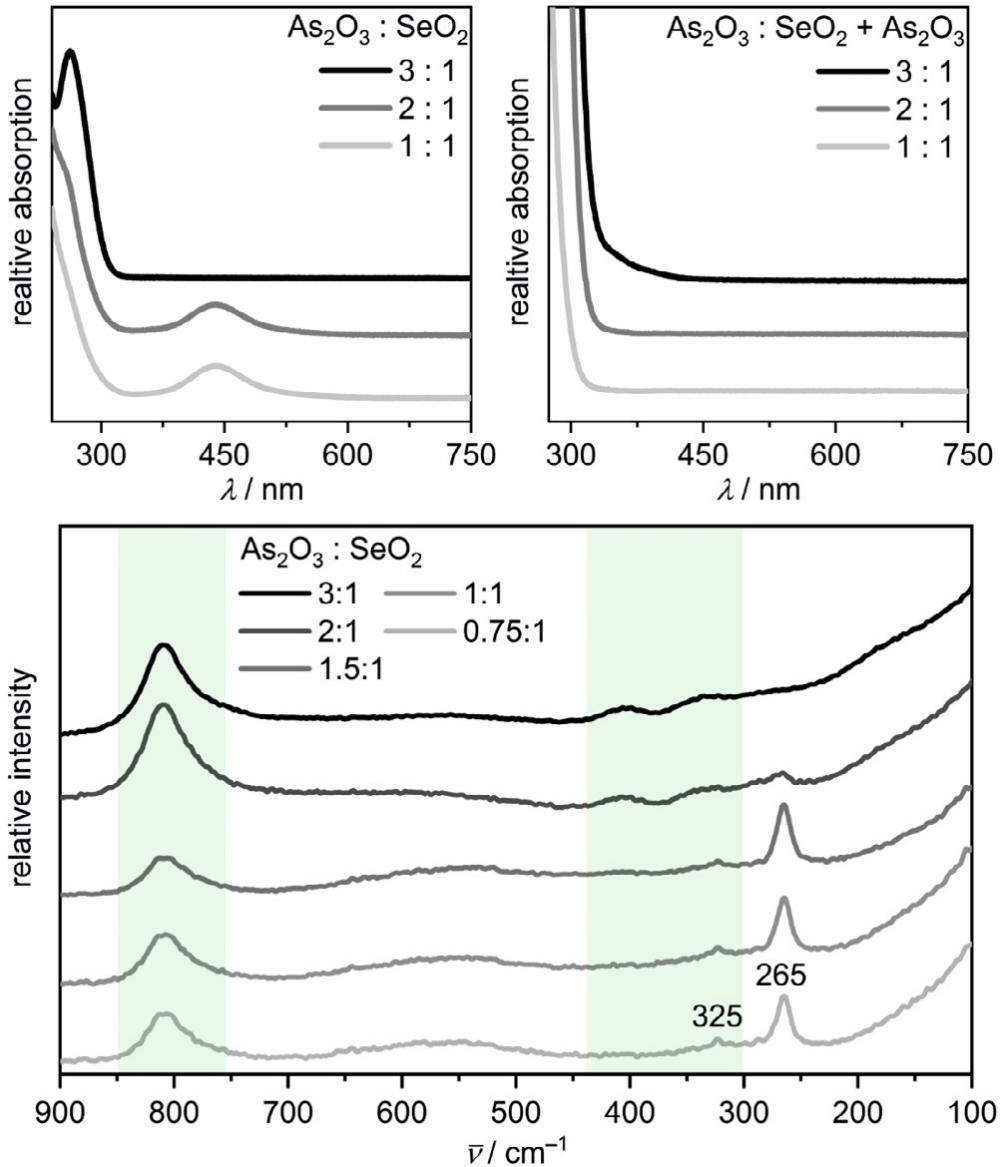
UV/Vis (top left) and Raman (bottom) spectra of selenide solutions synthesized with different *q*(Se) ratios. Absorption spectra of selenide solutions after adding dissolved As_2_O_3_ at room temperature are presented (top right). The green boxes mark the wavenumber range, where As−O vibrations of the AsO_4_^3−^ anion occur.

In low‐concentrated alkaline solutions, the optical absorption band of the diselenide anion Se_2_^2−^ had been reported at about 430 nm.[^19^, ^20^, ^23^] Se_3_^2−^ and Se_4_^2−^ absorb at 530 nm and 470 nm, respectively.^[19]^ The UV/Vis spectrum (Figure 5) of an orange solution synthesized with *q*(Se)=1 in hydroflux exhibited a symmetrical absorption band with its maximum at 440 nm, which we attribute to Se_2_^2−^ with respect to the above cited literature. For higher *q*(Se) ratios, viz. 2 and 3, the formation of the monochalcogenide anion was observed in the UV/Vis, similar as in the case of the tellurides.

Raman spectra of dissolved oligoselenides are scarce. In acetone, the Se_6_^2−^ anion exhibits vibrations bands at 235 cm^−1^, 285 cm^−1^ and 405 cm^−1^.^[39]^ In low‐concentrated alkaline solutions, the oxidation of H_2_Se with H_2_O_2_ had yielded selenium species with average oxidations states of −1, −0.67 and −0.5, that is, Se_*n*_^2−^ (*n*=2, 3, 4), and Raman bands at 269 cm^−1^ and 324 cm^−1^.^[22]^ The band at 269 cm^−1^ had been assigned to the Se_4_^2−^ anion based on the Raman resonance of a measurement with a 476 nm laser,^[22]^ since the Se_4_^2−^ anion has an absorption band at 470 nm.^[19]^ A Raman spectrum measured with an 457 nm laser had resulted in an even greater intensity of the 269 cm^−1^ band.^[22]^ DFT calculations had predicted two Raman active vibration modes at 299 cm^−1^ and 106 cm^−1^ for the Se_4_^2−^ anion and one Raman band at 273 cm^−1^ for Se_2_^2−^.^[22]^ In glassy selenium, the Se−Se stretching mode had been reported to occur at 250 cm^−1^ with a shoulder at 235 cm^−1^,^[44]^ which is similar to As−Se (238 cm^−1^, 252 cm^−1^)^[45]^ and Se−Te glasses (220 cm^−1^ to 280 cm^−1^).^[41]^

The Raman spectra (Figure 5) of solutions obtained from hydroflux reactions with *q*(Se) of 0.75, 1, or 1.5 show a vibration band with a maximum at 265 cm^−1^, which could indicate higher oligoselenides, although the transmission UV/Vis spectra revealed exclusively Se_2_^2−^ for *q*(Se)=1. However, the concentration in the optical spectroscopy had to be about 30 times lower than for the Raman measurements when using standard quartz cuvettes. To exclude a concentration dependent product formation, we measured an UV/Vis spectrum in reflection mode on the same selenide solution used in the Raman spectroscopy, which showed only one absorption band at 435 nm confirming the presence of mainly Se_2_^2−^ anions (Figure S12, Supporting Information). Therefore and because of the absence of additional vibration bands^[22]^ we assign the 265 cm^−1^ band in the Raman spectra of the experiments with *q*(Se)=0.75, 1, or 1.5 to the diselenide anion Se_2_^2−^, which is also in good agreement with the calculated value of 273 cm^−1^.^[22]^

In all of our experiments, the diselenide band at 265 cm^−1^ was accompanied by a tiny band at 323 cm^−1^, which was proposed to be caused by Se_2_^−^.^[22]^ Both vibration bands had always the same intensity ratio, despite different *q*(Se). It had been stated that the Se_2_^−^ radical forms under intense laser light from oligoselenides Se_*n*_^2−^ with *n*=2–4.[^19^, ^22^] However, Se_2_^2−^ is an unlikely precursor, as an electron would have to be abstracted. Moreover, the excitation of Se_2_^2−^ with a 530 nm laser would be very inefficient because of its absorption band at 440 nm. The most plausible precursor for Se_2_^−^ is Se_4_^2−^, since its decomposition involves a symmetrical bond cleavage and its absorption band at 470 nm is close to the wavelength of the laser.[^19^, ^22^] The dissociation of Se_3_^2−^ would result in the Se^−^ radical besides Se_2_^−^, which had been observed in aqueous solutions.^[46]^ In our experiments, the presence of small amounts of other oligoselenides than Se_2_^2−^ can be explained by Equation (2) and (3). In addition, the Se_2_^−^ radical is known to have an absorption band between 490 and 520 nm[^47^, ^48^, ^49^] leading to an intensity enhancement of its vibration band at 323 cm^−1^ by Raman resonance, so that the actual Se_2_^−^ radical concentration is expectedly low. When changing the radiation source to a 1064 nm laser, the vibrations band at 323 cm^−1^ vanishes, while the Se_2_^2−^ band remains with no change in intensity (Figure S13, Supporting Information).

In the UV/Vis spectrum of the experiment with *q*(Se)=3 (Figure 5), the monoselenide anion Se^2−^ exhibits one absorption band at 262 nm, which is close to the reported value of 270 nm.[^19^, ^20^] The same result was obtained for a solution with higher concentration of the reactants, which were used for Raman spectroscopy (Figure S14, Supporting Information). The Raman spectra confirmed the absence of species with Se−Se bond.

By adding at room temperature a hydroflux solution of As_2_O_3_ with the same *q*(K) to an orange‐colored diselenide solution with *q*(Se)=1, the color and the absorption band of the Se_2_^2−^ anion vanishes. In this case, the strong absorption of the excess AsO_3_^3−^ ions below 300 nm overlays the signal of the monoselenide anion Se^2−^ (Figure S6, Supporting Information).

As the vibration bands of AsO_4_^3−^ and SeO_3_^2−^ overlap, we also used Sb_2_O_3_ as reducing agent. The reacted solution with *n*(Sb_2_O_3_):*n*(SeO_2_)=0.75 showed the Raman band of Se_2_^2−^ at 265 cm^−1^ (Figure S15, Supporting Information). The additional vibrational band at 810 cm^−1^ is in good agreement with the Raman spectrum of SeO_2_ dissolved under hydroflux conditions. The coexistence of SeO_3_^2−^ and Se_2_^2−^ anions confirmed the observation that no elemental selenium was formed in our experiments.

**Figure 6:**
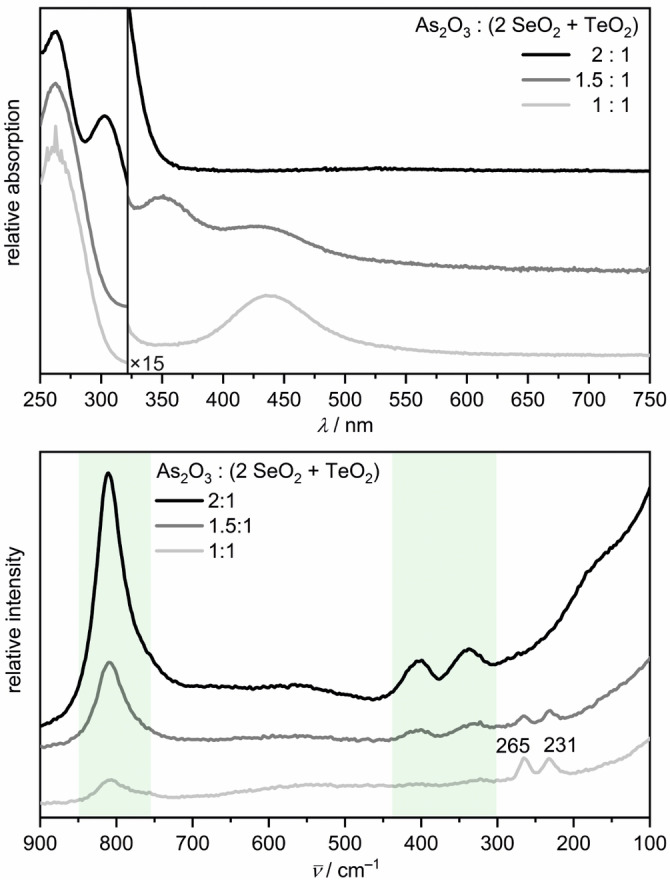
UV/Vis (top) and Raman (bottom) spectra of chalcogenide solutions synthesized with different *q*(SeTe) ratios. The green boxes mark the wavenumber range, where As−O vibrations of the AsO_4_^3−^ anion occur.

The heterochalcogenide solutions were prepared by starting from SeO_2_ and TeO_2_ in the molar ratio 2:1, according to the molar fractions of the chalcogens in K_2_Se_2_Te. As_2_O_3_ was added as reducing agent in 1, 1.5 and 2 equivalents, i.e., *q*(SeTe)=As_2_O_3_:(^2^/_3_ SeO_2_+^1^/_3_ TeO_2_). The UV/Vis spectrum of the experiment with *q*(SeTe)=1 shows the Se_2_^2−^ band at 435 nm as well as the Se^2−^ band at 262 nm (Figure 6), while in the spectrum for *q*(SeTe)=1.5, one additional band appears at 351 nm. There is very little spectroscopic information on mixed chalcogenides that could be used for comparison. NMR experiments on the mixed chalcogenides (TeSe_2_)^2−^ and (TeSe_3_)^2−^ in ethylenediamine revealed several equilibria between those anions and tellurium richer phases, for example, (Te_2_Se)^2−^ and (Te_3_Se)^2−^.^[50]^ In analogy, we propose a similar equilibrium between the diselenide and ditelluride anions [Eq. 6]. Consequently, the absorption band at 351 nm is assigned to the (SeTe)^2−^ anion, which was not described before.

(6) $$\mathrm{Se}{}_{2}{}^{2-}\;+\;\mathrm{Te}{}_{2}{}^{2-}\;\vec{\leftarrow }\;2\;\left(\mathrm{SeTe}\right){}^{2-}$$

The Raman spectra (Figure 6) for *q*(SeTe)=1 and 1.5 show the Se_2_^2−^ band at 265 cm^−1^ and one additional band at 231 cm^−1^, which occurs in the typical energy range of Se−Te vibrations and is therefore assigned to the (SeTe)^2−^ anion.[^41^, ^51^, ^52^, ^53^, ^54^] The Se−Te stretching vibrations in Se−Te glasses had been reported to occur between 205 cm^−1^ and 216 cm^−1^.[^52^, ^53^] A similar shift to lower energies had been observed for Se−Se and Te−Te vibrations in the solid state compared with Se_2_^2−^ and Te_2_^2−^ anions in aqueous solutions. DFT calculations on mixed trichalcogenides *Ch*_3_^2−^ (*Ch*=Se, Te) had predicted a range from 218 cm^−1^ to 230 cm^−1^ for Se−Te stretching modes.^[41]^ In [Zn(NH_3_)_4_](TeSe_3_), they occur at 217 cm^−1^ and 231 cm^−1^,^[54]^ in Na_2_TeSe_3_ at 213 cm^−1^ and 238 cm^−1^.^[51]^

The vibrations bands of the Se_2_^2−^ and the (SeTe)^2−^ anions are clearly detectable. The Se_2_^2−^ band is the more intense for *q*(SeTe)=1, but the weaker for *q*(SeTe)=1.5. As indicated by the Raman spectra, TeO_2_ is only partly reduced, as small intensities of Te−O vibrations of the TeO_3_^2−^ anion are visible with *q*(SeTe)=1 (Figure S16, Supporting Information). The assumption of (SeTe)^2−^ anions that formed from a 2:1 solution of SeO_2_ and TeO_2_ is indirectly corroborated by the remaining Se_2_^2−^anions visible in the Raman spectrum. The Raman spectrum of the experiment with *q*(SeTe)=1.5 has an overall lower intensity of the chalcogenide vibration bands than the one with *q*(SeTe)=1, since the crystallization of K_2_Se_2_Te has lowered the concentration of dissolved chalcogenide anions.

The UV/Vis spectrum of the experiment with *q*(SeTe)=2 shows the absorption bands of Se^2−^ at 261 nm and Te^2−^ at 305 nm, indicating that the high amount of As_2_O_3_ had reduced the chalcogenide(IV) oxides completely. Accordingly, no Raman band is found in the range of *Ch*−*Ch* vibrations.

When mixing pre‐synthesized Se_2_^2−^ and Te_2_^2−^ solutions at room temperature, the resulting mixture shows a strong (SeTe)^2−^ band at 231 cm^−1^ and a smaller Se_2_^2−^ band at 265 cm^−1^ (Figure S17, Supporting Information). The crystallization of K_2_Te_3_ had reduced the Te_2_^2−^ concentration, while the concentration was too low for the precipitation of K_2_Se_3_. This experiment corroborates the equilibrium in Equation (6).

The chalcogenide solutions were sensitive against atmosphere. The colorless monotelluride solutions started to oxidize on the slightest contact with oxygen resulting in purple solutions containing Te_2_^2−^, from which then elemental tellurium precipitated. The latter can be identified by Te−Te vibration bands at 120 cm^−1^ and 139 cm^−1^ (Figure S18, Supporting Information).^[55]^ The reaction takes place on the surface of the liquid, which allows handling them in air for a short period. Upon adding As_2_O_3_ solution, the tellurium is again reduced to tellurides (Figure S19, Supporting Information).

The selenide solutions are less reactive. Monoselenide solutions showed the orange color of Se_2_^2−^ only after several hours in air. When diselenide solutions were diluted with water and exposed to air, a red film initially formed on the surface of the liquid, which yielded a grey powder after several hours that showed Se‐Se vibrations bands at 140 cm^−1^ and 235 cm^−1^ (Figure S18, Supporting Information).[^56^, ^57^] Higher oligochalcogenides could not be detected.

Experiments concerning the reductive potential of As and As_2_O_3_ yielded unexpected results, e.g., that elemental arsenic is unable to reduce *Ch*_2_^2−^ to *Ch* ^2−^, and that excess As^III^O_3_^3−^ anions seem to disproportionate into arsenic and As^V^O_4_^3−^ (see Supporting Information).

## Conclusion

Solutions of mono‐ and dichalcogenide anions *Ch* ^2−^, *Ch*_2_^2−^ (*Ch*=Se, Te) and (SeTe)^2−^ are accessible by reducing the respective *Ch*O_2_ with As_2_O_3_ under hydroflux conditions. When a sub‐stoichiometric amount of As_2_O_3_ is added, the reaction product consists of a mixture of *Ch*O_3_^2−^ and *Ch*_2_^2−^ anions. However, neither elemental chalcogen nor oligochalcogenide anions larger than *Ch*_2_^2−^ were observed spectroscopically. The addition of As_2_O_3_ in excess yields colorless solutions of monochalcogenide anions *Ch* ^2−^. Large single‐crystals of K_2_ *Ch*_3_ were obtained when the amount of As_2_O_3_ does not allow an average oxidation state of the chalcogen that is more negative than −0.67 (*Ch*_3_^2−^). The crystallization of K_2_Se_3_ or K_2_Se_2_Te requires higher reactants concentrations than needed for K_2_Te_3_.

The preparation of chalcogenides via the hydroflux method represents an attractive alternative to the hitherto used synthesis routes. The necessary equipment is cheaper and the procedure is simpler and also safer. The unexpected formation of chalcogenides from chalcogen dioxides is due to the ultra‐alkaline conditions. The water in the reaction mixture is strongly bonded to hydroxide. Its reduced activity prevents the hydrolysis of the trichalcogenides, but also shifts the redox equilibria known from dilute alkaline solutions.^[24]^ A transfer of the approach to other systems should be possible.

## Conflict of interest

The authors declare no conflict of interest.

## Supporting information

As a service to our authors and readers, this journal provides supporting information supplied by the authors. Such materials are peer reviewed and may be re‐organized for online delivery, but are not copy‐edited or typeset. Technical support issues arising from supporting information (other than missing files) should be addressed to the authors.

Supporting Information
