# Supplementary material for: Chalcogenides by Reduction of their Dioxides in Ultra‐Alkaline Media
Source: Angew Chem Int Ed Engl. 2021 Sep 6;60(41):22570–7. doi: 10.1002/anie.202107642 (PMC8518872; doi:10.1002/anie.202107642)
Supplement: Supplementary file 1 — Supporting Information [file ANIE-60-22570-s001.pdf]

Angewandte Chemie
Eine Zeitschrift der Gesellschaft Deutscher Chemiker

Supporting Information

Chalcogenides by Reduction of their Dioxides in Ultra-Alkaline Media

*Ralf Albrecht and Michael Ruck**

anie_202107642_sm_miscellaneous_information.pdf

Table of Contents

Reductive Potential of As and As_2O_3 2
Experimental Procedures 3
Results and Discussion 5
Powder X-ray Diffraction (PXRD) Analysis....................5
Energy Dispersive X-ray (EDX) Spectroscopy Data....................7
Crystal Structure Information....................8
Spectroscopic Characterization....................11
References 21
Author Contribution....................21

Reductive Potential of As and As_2O_3

In all of our experiments with an excess of As_2O_3 as reducing agent, i.e., $q(Ch) \geq 2$, the Raman and UV-Vis spectra never indicated the presence of residual $As^{III}O_3^{3-}$. In these experiments, a black powder was found at the bottom of the PTFE inlet. After isolation and washing with water, the powder was identified as elemental arsenic by PXRD (Figure S20, Supporting Information). The excess in $As^{III}O_3^{3-}$ anions seems to disproportionate into arsenic and $As^{V}O_4^{3-}$ (Equation S1).

$$5\ As^{III}O_3^{3-} + 3\ H_2O \rightarrow 2\ As^0 + 3\ As^{V}O_4^{3-} + 6\ OH^- \qquad (S1)$$

However, experiments in which As_2O_3 was dissolved in a hydroflux without the addition of chalcogen dioxides showed no evidence of $As^{V}O_4^{3-}$ anions or elemental arsenic (Figure S8, Supporting Information). The chalcogenide anions seem to catalyze the disproportion of the $As^{III}O_3^{3-}$ anions.

Upon replacing As_2O_3 by elemental arsenic, the reduction of ChO_2 to dichalcogenide anions Ch_2^{2-} still proceeded under hydroflux conditions (Figure S21 and S22, Supporting Information). However, even with a high excess of arsenic neither the color of these solutions vanished nor was the intensity of the *Ch*–*Ch* vibration band decreased significantly. UV-Vis measurements of these solutions revealed only a small fraction of monochalcogenide anions (Figure S23 and S24, Supporting Information). Similar observations were made when the reaction time was extended to 48 h. Obviously, elemental arsenic is not able to reduce Ch_2^{2-} to Ch^{2-} under these conditions. The small amount of monochalcogenide anions developed during the reaction is probably caused by intermediately formed $As^{III}O_3^{3-}$ (Equation S2), as the latter can not only reduce ChO_3^{2-} but also Ch_2^{2-}. In summary, elemental arsenic is able to reduce ChO_3^{2-} to Ch_2^{2-}, but not Ch_2^{2-} to Ch^{2-}, which is achieved by $As^{III}O_3^{3-}$ anions even at room temperature.

$$10\ As^0 + 6\ ChO_3^{2-} + 24\ OH^- \rightarrow 10\ As^{III}O_3^{3-} + 3\ Ch_2^{2-} + 12\ H_2O \qquad (S2)$$

In additional experiments, we reacted elemental selenium or tellurium with As. In the course of preparation of the hydroflux reaction mixtures, first water was added to elemental selenium or tellurium and then solid KOH, which results in a temperature rise. After few minutes, a color change was observed. Raman spectra confirmed the formation of Ch_2^{2-} anions (Figure S25, Supporting Information). The products formed after heating to 200 °C where the same as in reactions starting from ChO_2 (Figure S26, Supporting Information).

Experimental Procedures

Synthesis

The chalcogenide syntheses were performed in a KOH (86 %, Fischer Scientific) hydroflux with water-base ratio of 1.9. These reactions were carried out in a PTFE-lined 50 mL Berghof type DAB-2 autoclave to prevent water loss. The following starting materials were used as purchased: Se (99.5 %, Alfa Aesar), SeO_2 (99.8 %, abcr), Te (99.999%, ChemPur), TeO_2 (99.9 %, abcr), As (99 %, abcr), As_2O_3 (99.996 %, abcr), As_2O_5 (99.9 %, abcr), V_2O_3 (95 %, abcr) V_2O_5 (99.5 %, Riedel-de Haën), Sb_2O_3 (99.6 %, abcr), Sb_2O_5 (99.999 %, abcr). The starting materials for the respective synthesis of K_2Se_3 (5 mmol SeO_2, 6 mmol As_2O_3), K_2Te_3 (2 mmol TeO_2, 2.4 mmol As_2O_3) and K_2Se_2Te (1.33 mmol SeO_2, 0.67 mmol TeO_2, 2.6 mmol As_2O_3) were dissolved/suspended in 3 ml deionized water before 5.7 g of KOH was added. After sealing the autoclave at ambient conditions, the reaction mixture was heated to 200 °C at 2 °C/min, held for 48 h before being cooled down to room temperature at a rate of 0.1 °C/min. In the case of K_2Se_2Te, a reaction duration of 10 h and a cooling rate of 1 °C/min yielded larger crystals. The products of the respective synthesis of K_2Se_3 (deep red solution, large black bars, about 2 cm), K_2Te_3 (pale purple solution, large black bars, about 1 cm) and K_2Se_2Te (deep brown solution, large intergrown plates, about 1 cm) were filtered under inert conditions by using a Schlenk-frit. The crystals were stored under argon.
The syntheses for the spectroscopic analyses were performed with the same hydroflux concentration. For the UV-Vis measurements, 0.2 mmol chalcogen reactant, 9 ml of deionized water and 17 g KOH was used, whereas for the Raman measurements 1 mmol, 3 ml H2O and 5.7 g KOH was used. The amount of reducing agent can be calculated based on the given $q(Ch)$ values. The sealed autoclave was heated to 200 °C at 2 °C/min, typically held for 5 h before being cooled down to room temperature at a rate of 1 °C/min. After the reaction, the products were prepared as fast as possible for the respective spectroscopic analysis.

Crystal Structure Determination

Diffraction data were collected at 100(1) K with a four-circle diffractometer Kappa Apex2 (Bruker) equipped with a CCD-detector using graphite-monochromated Mo-Kα radiation (λ = 71.073 pm). The raw data were corrected for background, Lorentz and polarization factors,[1] and multi-scan absorption correction was applied.[2] The structures were solved using ShelXT.[3] Structure refinement against F^2 with ShelXL[4] included anisotropic displacement parameters for all atoms. All graphical representations of the structure were developed with Diamond.[5] Table S2 to S8 of the Supporting information contain crystal structure data. Further details of the crystal structure determination are available from the Fachinformationszentrum Karlsruhe, D-76344 Eggenstein-Leopoldshafen (Germany), E-mail: crysdata@fiz-karlsruhe.de, on quoting the depository number listed in Table S2 for K_2Se_3, K_2Te_3 and K_2Se_2Te.

Powder X-ray Diffraction

X-ray powder diffraction patterns for phase identification were measured at room temperature on an Empyrean (Pananalytical) equipped with a curved Ge(111) monochromator using Cu-Kα_1 radiation (λ = 154.056 pm).

Raman Spectroscopy

Raman spectra were recorded using a DXR SmartRaman spectrometer (Thermo Scientific) equipped with a 532 nm Laser and CCD detector working with a measuring range of 3500 to 30 cm^{-1}. In addition, the RFS 100 FT-Raman spectrometer (Bruker) was used, which was equipped with a 1064 nm laser and Ge detector.

UV-Vis Spectroscopy

The transmission spectra were recorded using a Cary50 (Varian) UV-Vis spectrometer in a measuring range from 200 nm to 800 nm. The samples were analyzed with a standard quartz glass cuvette. When the maximum absorbance of the instrument was reached, the sample was diluted with a KOH hydroflux q(K) = 1.9. Samples with reactant concentrations used for Raman measurements were characterized with diffuse reflection at the same

instrument. One droplet of the sample was placed between two quartz glass plates and the detector was positioned above.

SEM and EDX Analysis

Scanning electron microscopy (SEM) was performed using a SU8020 (Hitachi) with a triple detector system for secondary and low-energy backscattered electrons (U_a = 5 kV). The composition of selected crystals was determined by semi-quantitative energy dispersive X-ray analysis (U_a = 15 kV) using a Silicon Drift Detector (SDD) X-MaxN (Oxford Instruments). The data were processed (integration, pulse-pile up correction) applying the AZtec software package (Oxford Instruments, 2013).

Results and Discussion

Powder X-ray Diffraction (PXRD) Analysis

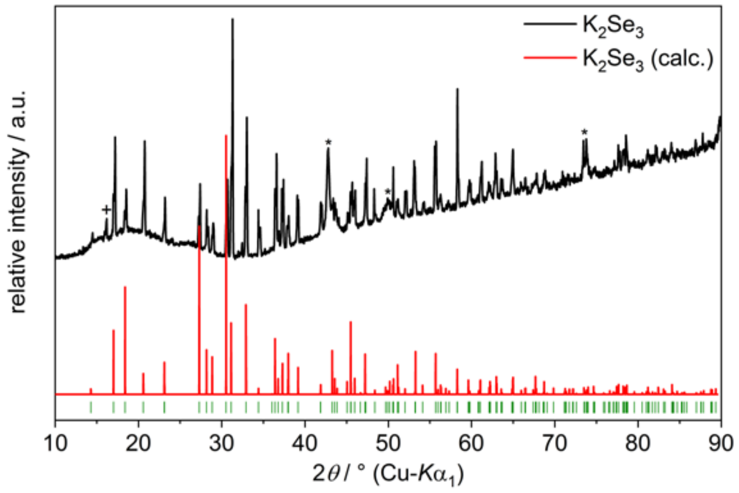

Figure S1. Powder X-ray diffractogram of K_2Se_3 measured with an air-sensitive sample holder equipped with Kapton foil causing the large background from 10 to 30° 2*θ*. Reflections marked with an asterisk indicate the background of the sample holder, see Figure S4. One reflection is marked with a plus indicating KOH as by-product.

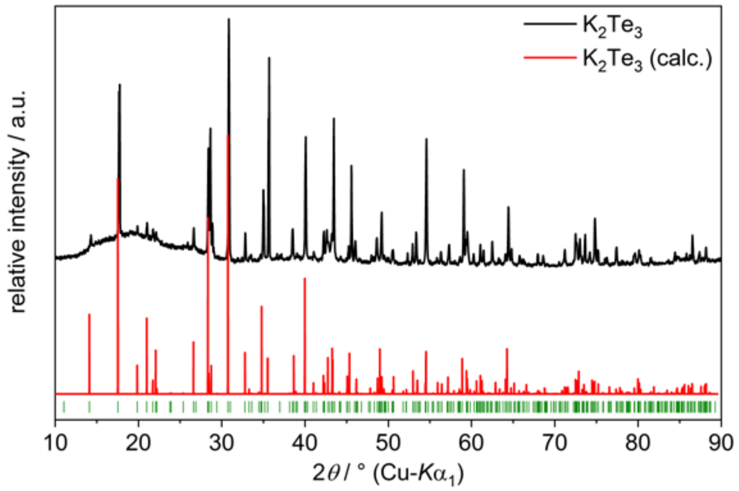

Figure S2. Powder X-ray diffractogram of K_2Te_3 measured with an air-sensitive sample holder equipped with Kapton foil causing the large background from 10 to 30° 2*θ*.

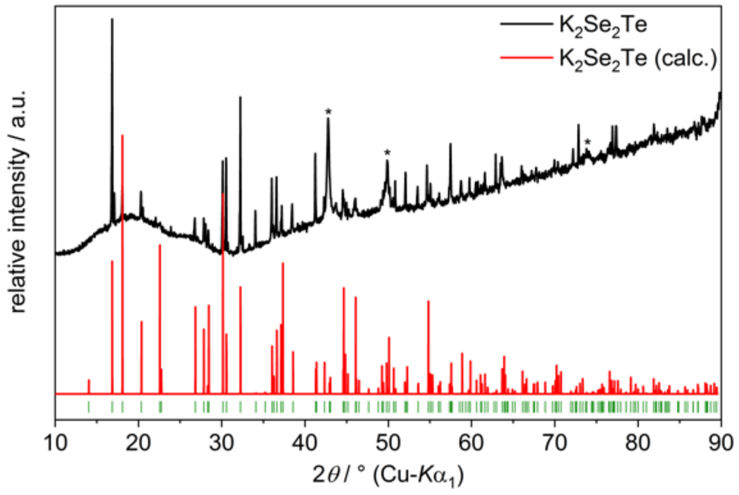

Figure S3. Powder X-ray diffractogram of K_2Se_2Te measured with an air-sensitive sample holder equipped with Kapton foil causing the large background from 10 to 30° 2*θ*. Reflections marked with an asterisk indicate the background of the sample holder, see Figure S4.

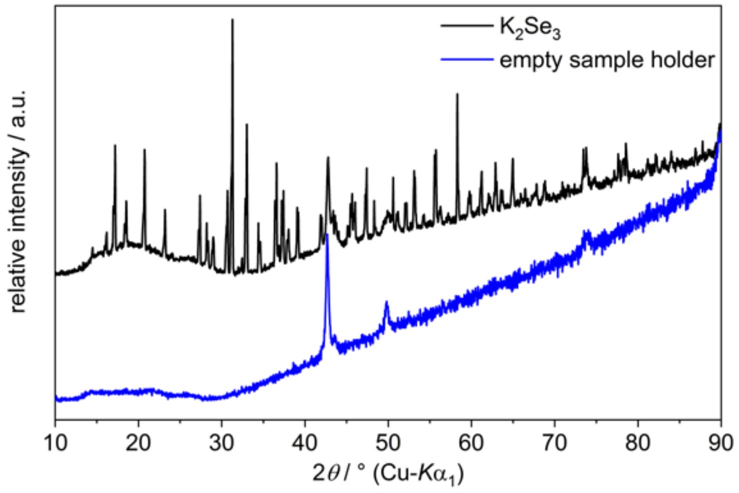

Figure S4. Powder X-ray diffractogram of K_2Se_3 compared with a measurement of an empty air-sensitive sample holder.

Energy Dispersive X-ray (EDX) Spectroscopy Data

Table S1. Results of the energy-dispersive X-ray spectroscopy (EDX) of K_2Se_3, K_2Te_3 and K_2Se_2Te. EDX analyses of several crystals were averaged. The standard deviations (in brackets) do not include systematic errors.

	K_2Se_3		K_2Te_3		K_2Se_2Te		
	K	Se	K	Te	K	Se	Te
theoretical	2	3	2	3	2	2	1
measured	1.9(1)	3.0(1)	2.0(1)	3.0(1)	2.0(1)	2.2(1)	1.0(1)

Crystal Structure Information

Table S2. Crystal data and structure refinement of K_2Se_3, K_2Te_3 and K_2Se_2Te.

Crystal data	K_2Se_3	K_2Te_3	K_2Se_2Te
Crystal system, space group (no.)	orthorhombic, $Cmc2_1$ (36)	orthorhombic, *Pnma* (62)	orthorhombic, $Cmc2_1$ (36)
Temperature / K	100(1)	100(1)	100(1)
Radiation, wavelength	Mo K_α, 71.073 pm	Mo K_α, 71.073 pm	Mo K_α, 71.073 pm
Unit cell / pm	*a* = 765.75(9)<br>*b* = 1036.5(1)<br>*c* = 764.57(9)	*a* = 1591.45(9)<br>*b* = 1005.47(6)<br>*c* = 463.29(3)	*a* = 783.42(4)<br>*b* = 1045.64(6)<br>*c* = 777.13(4)
Z	8	4	8
Volume / (10^6·pm^3)	606.8(1)	741.3(1)	636.6(1)
Density (calc.) / (g·cm^{-3})	3.45	4.13	3.80
$2\theta_{max}$ (MoK_α) / °	90	90	90
Independent reflections	2593	3166	2723
No. of parameters	30	43	28
R_{int}, R_σ	0.034, 0.026	0.042, 0.033	0.039, 0.034
$R_1[F>2\sigma(F)]$, $wR_2(F^2)$	0.017, 0.029	0.024, 0.032	0.023, 0.049
GooF(F^2)	1.026	1.063	1.065
Residual electron density / (e·10^{-6} pm^{-3})	+0.55 to −0.74	+1.37 to −1.25	+1.42 to −1.20
CSD number	2084650	2084652	2084651

Table S3. Atomic coordinates and equivalent isotropic displacement parameters (/ pm²) in K_2Se_3 at 100(1) K.

	Wyckoff symbol	*x*	*y*	*z*	U_{iso}/U_{eq}
Se1	8*b*	0.25829(2)	0.34019(2)	0.00001(2)	75(1)
Se2	4*a*	1/2	0.29019(2)	0.18389(3)	66(1)
K1	4*a*	0	0.10003(4)	0.12646(7)	91(1)
K2	4*a*	0	0.41742(5)	0.34623(6)	100(1)

Table S4. Anisotropic displacement parameters (/ pm²) in K_2Se_3 at 100(1) K.

	U11	*U22*	*U33*	*U23*	*U13*	*U12*
Se1	60(1)	84(1)	82(1)	6(1)	–6(1)	–1(1)
Se2	67(1)	68(1)	64(1)	4(1)	0	0
K1	101(2)	69(2)	102(2)	1(1)	0	0
K2	114(2)	96(2)	89(2)	–7(1)	0	0

Table S5. Atomic coordinates and equivalent isotropic displacement parameters (/ pm²) in K_2Te_3 at 100(1) K.

	Wyckoff symbol	*x*	*y*	*z*	U_{iso}/U_{eq}	Occupancy
Te1(a)	4*c*	0.12243(2)	1/4	0.17770(1)	65(1)	0.975(1)
Te1(b)	4*c*	0.1362(5)	1/4	0.322(2)	219(14)	0.025(1)
Te2(a)	4*c*	0.25988(2)	1/4	0.79562(4)	73(1)	0.975(1)
Te2(b)	4*c*	0.2765(6)	1/4	0.695(2)	219(14)	0.025(1)
Te3(a)	4*c*	0.48167(2)	1/4	0.68901(4)	70(1)	0.975(1)
Te3(b)	4*c*	0.4981(6)	1/4	0.802(2)	219(14)	0.025(1)
K(a)	8*d*	0.37745(3)	0.02967(4)	0.21246(9)	111(1)	0.975(1)
K(b)	8*d*	0.363(1)	0.025(2)	0.275(4)	111(1)	0.025(1)

Table S6. Anisotropic displacement parameters (/ pm²) in K_2Te_3 at 100(1) K.

	U11	*U22*	*U33*	*U12*	*U13*	*U23*
Te1(a)	0.00632(5)	0.00642(5)	0.00677(7)	0	–0.00039(5)	0
Te1(b)	0.019(2)	0.026(2)	0.021(3)	0	–0.005(2)	0
Te2(a)	0.00576(6)	0.00814(6)	0.00786(6)	0	-0.00032(5)	0
Te2(b)	0.019(2)	0.026(2)	0.021(3)	0	–0.005(2)	0
Te3(a)	0.00577(6)	0.00792(6)	0.00736(6)	0	0.00036(5)	0
Te3(b)	0.019(2)	0.026(2)	0.021(3)	0	–0.005(2)	0
K(a)	0.0131(2)	0.0084(1)	0.0119(1)	0.0019(1)	–0.0023(1)	–0.0009(1)
K(b)	0.0131(2)	0.0084(1)	0.0119(1)	0.0019(1)	–0.0023(1)	–0.0009(1)

Table S7. Atomic coordinates and equivalent isotropic displacement parameters (/ pm²) in K_2Se_2Te at 100(1) K.

	Wyckoff symbol	*x*	*y*	*z*	U_{iso}/U_{eq}
Te	4*a*	1/2	0.21925(2)	0.21917(3)	57(1)
Se	8*b*	0.25389(3)	0.15579(3)	0.41870(4)	72(1)
K1	4*a*	0	0.07861(9)	0.08132(11)	100(1)
K2	4*a*	0	0.39650(8)	0.28065(11)	89(1)

Table S8. Anisotropic displacement parameters (/ pm²) in K_2Se_2Te at 100(1) K.

	U11	*U22*	*U33*	*U23*	*U13*	*U12*
Se1	0.00553(6)	0.00566(7)	0.00577(7)	–0.00002(7)	0	0
Se2	0.00560(8)	0.00864(9)	0.00737(9)	–0.00059(7)	0.00107(7)	–0.00011(7)
K1	0.0123(3)	0.0093(3)	0.0084(3)	–0.0005(2)	0	0
K2	0.0105(3)	0.0070(3)	0.0092(3)	–0.0004(2)	0	0

Table S9. Selected bond lengths and angles in K_2Se_3, K_2Se_2Te and K_2Te_3 at 100(1) K (*Ch* = Se, Te).

	K_2Se_2Te	K_2Se_3	K_2Te_3
Ch–*Ch* / pm	256.2(1)	238.1(1)	281.2(1)<br>281.4(1)
Ch–*Ch*–*Ch* / °	97.6(1)	102.0(1)	103.8(1)
K1–*Ch* / pm	2× 338.9(1)<br>2× 340.0(1)<br>2× 360.9(1)<br>352.0(1), 390.7(1)	2× 332.3(1)<br>2× 340.8(1)<br>2× 345.9(1)<br>324.1(1), 357.0(1)	348.4(1), 354.0(1)<br>354.3(1), 358.2(1)<br>362.5(1), 367.8(1)<br>374.8(1), 396.3(1)
K2–*Ch* / pm	2× 338.3(1)<br>2× 345.4(1)<br>2× 349.6(1)<br>340.8(1), 361.6(1)	2× 340.0(1)<br>2× 340.7(1)<br>2× 345.5(1)<br>336.1(1), 405.8(1)	–
K–K / pm	366.7(1)	369.4(1)	441.0(1)

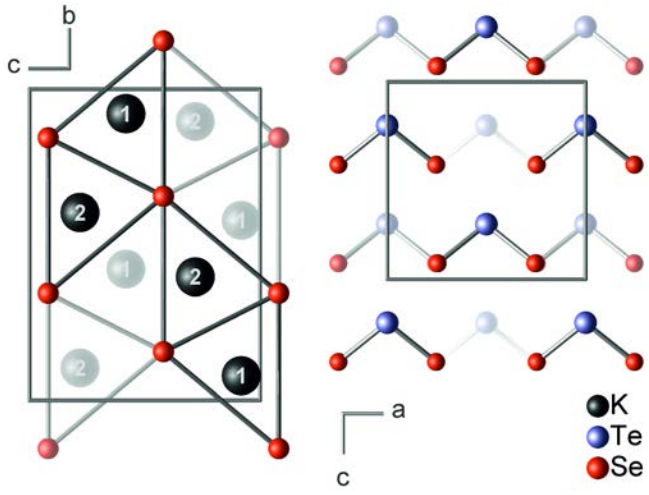

Figure S5. Arrangement of the $[KSe_6]^{5-}$ polyhedra viewing the (100) plane (left) and ordering of the corrugated $[TeSe_2]^{2-}$ layers with a AB stacking sequence viewing perpendicular to [010] (right) in the crystal structure of K_2TeSe_2.

Spectroscopic Characterization

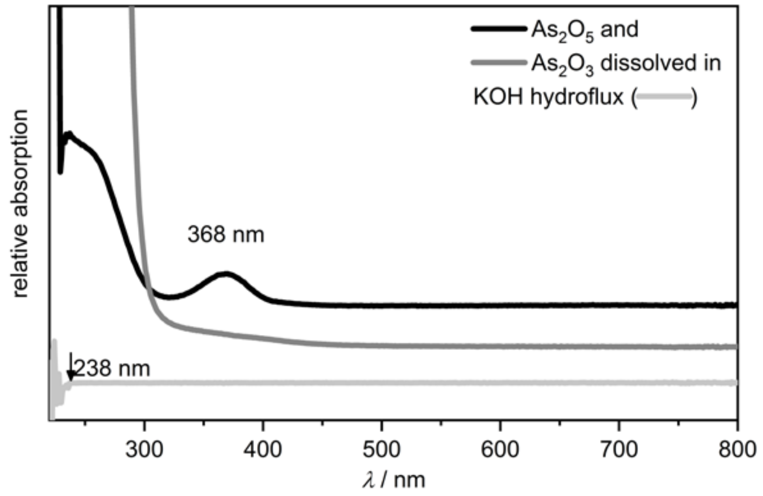

Figure S6. UV-Vis spectra of As_2O_3 and As_2O_5 dissolved in a potassium hydroxide hydroflux with *q*(K) = 1.9. These mixtures were heated up to 200 °C for 1 hour and measured after cooling to room temperature. The UV-Vis spectrum of the hydroflux has a bad resolution below 240 nm due to strong absorptions of the hydroflux itself.

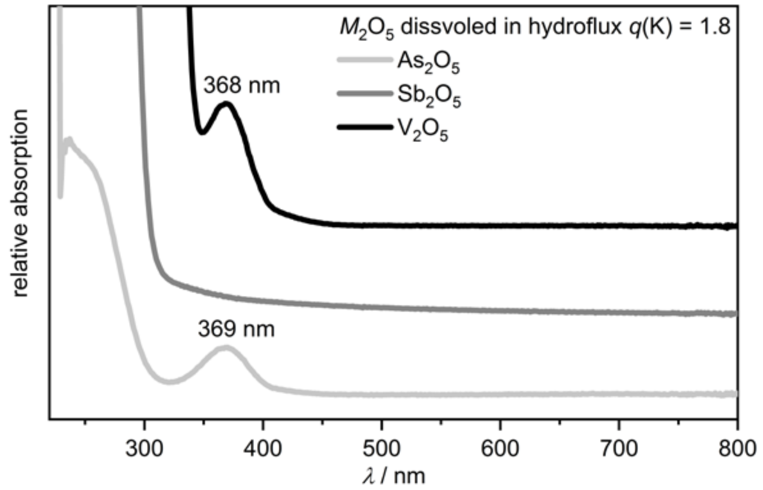

Figure S7. UV-Vis spectra of As_2O_5, Sb_2O_5 and V_2O_5 dissolved in a potassium hydroxide hydroflux with *q*(K) = 1.9. These mixtures were heated up to 200 °C for 1 hour and measured after cooling to room temperature. All three solutions have the same concentration.

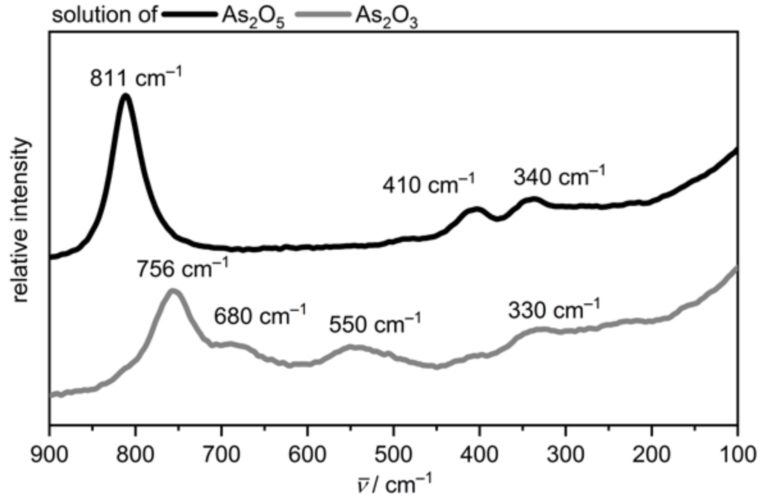

Figure S8. Raman spectra of As_2O_3 and As_2O_5 dissolved in a potassium hydroxide hydroflux with *q*(K) = 1.9. These mixtures were heated up to 200 °C for 1 hour and measured after cooling to room temperature.

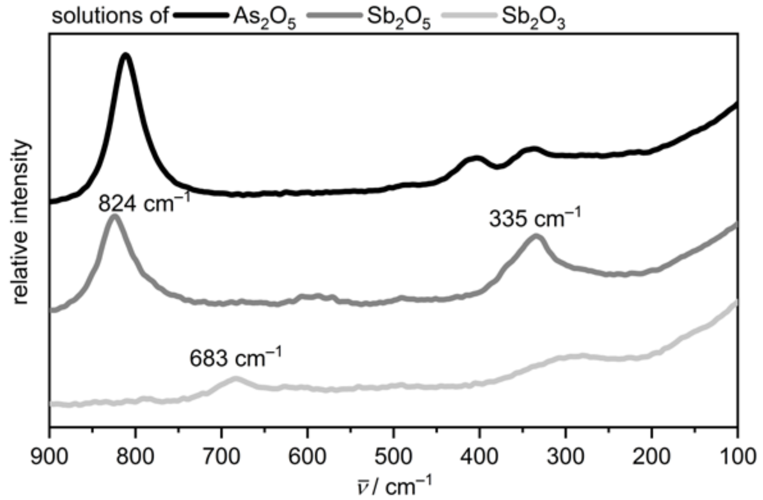

Figure S9. Raman spectra of Sb_2O_3 and Sb_2O_5 dissolved in a potassium hydroxide hydroflux with *q*(K) = 1.9 compared with the spectrum of dissolved As_2O_5. These mixtures were heated up to 200 °C for 1 hour and measured after cooling to room temperature.

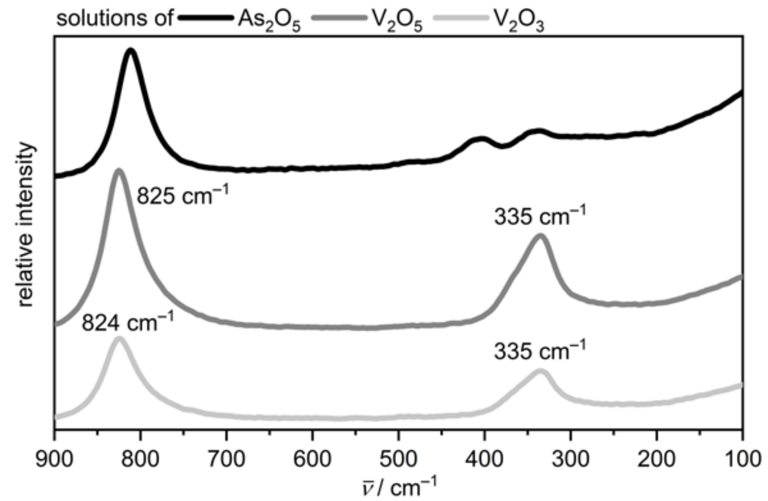

Figure S10. Raman spectra of V_2O_3 and V_2O_5 dissolved in a potassium hydroxide hydroflux with *q*(K) = 1.9 compared with the spectrum of dissolved As_2O_5. These mixtures were heated up to 200 °C for 1 hour and measured after cooling to room temperature.

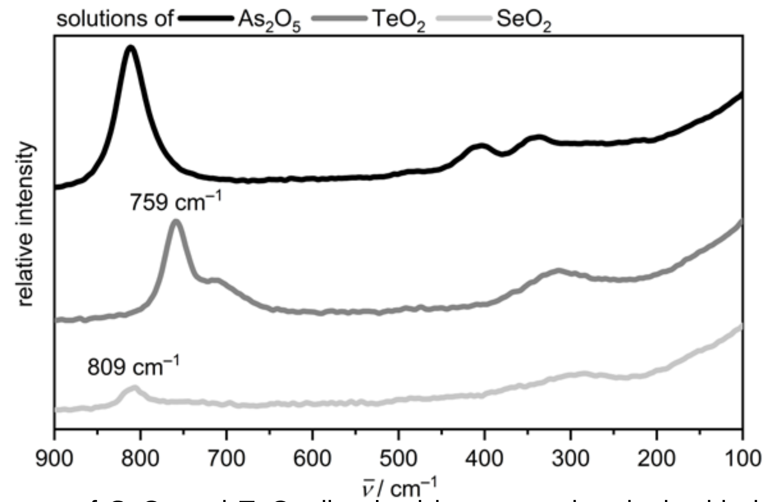

Figure S11. Raman spectra of SeO_2 and TeO_2 dissolved in a potassium hydroxide hydroflux with *q*(K) = 1.9 compared with the spectrum of dissolved As_2O_5. These mixtures were heated up to 200 °C for 1 hour and measured after cooling to room temperature.

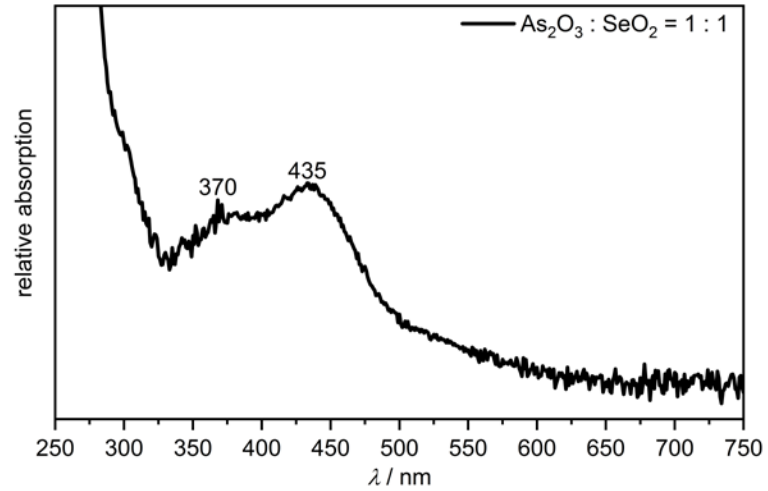

Figure S12. UV-Vis spectrum of an orange selenide solution synthesized with *q*(Se) = 1 in a potassium hydroxide hydroflux with *q*(K) = 1.9 with the same reactant concentration used for the Raman experiments. The UV-Vis spectrum was measured in reflection mode, where on droplet of the selenide solution was placed between two quartz glass plates. The band at 370 nm is caused by $As^{V}O_4^{3-}$ anions, see Figure S7.

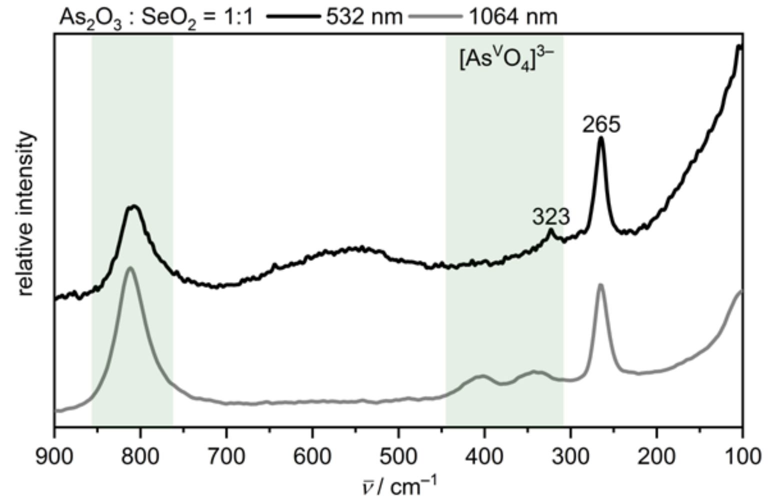

Figure S13. Raman spectra of an orange selenide solution synthesized with *q*(Se) = 1 in a potassium hydroxide hydroflux with *q*(K) = 1.9 measured with two different laser radiations.

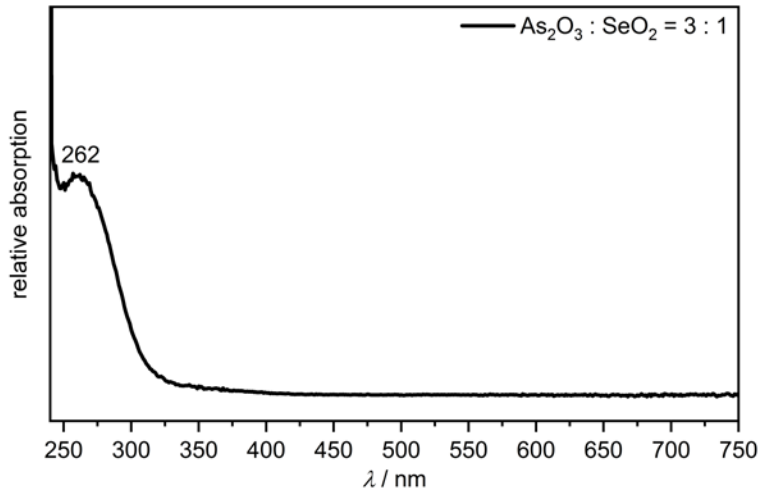

Figure S14. UV-Vis spectrum of an colorless selenide solution synthesized with *q*(Se) = 3 in a potassium hydroxide hydroflux with *q*(K) = 1.9 with the same reactant concentration used for the Raman experiments. The UV-Vis spectrum was measured in reflection mode, where on droplet of the selenide solution was placed between two quartz glass plates.

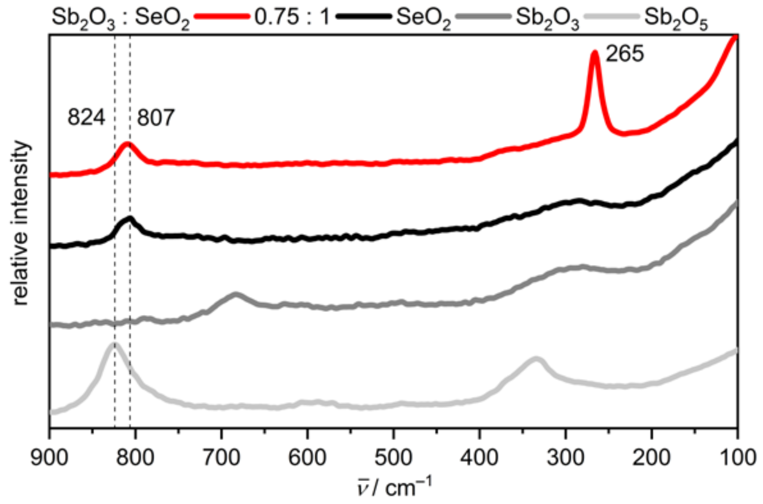

Figure S15. Raman spectrum of an orange solution synthesized by starting from a Sb_2O_3:SeO_2 ratio of 0.75.

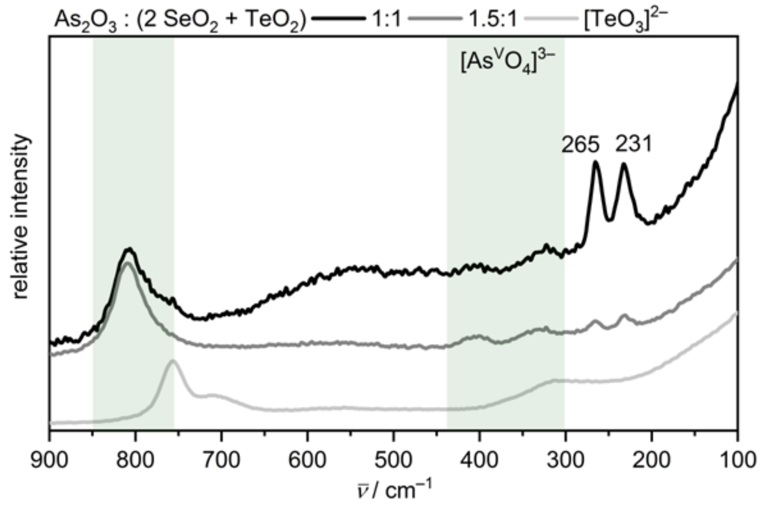

Figure S16. Raman spectra of mixed chalcogenide solutions obtained by hydroflux syntheses with different q(SeTe) ratios. The AsO_4^{3-} band at 811 cm^{-1} has a shoulder at lower wavenumbers indicating the existence of unreduced TeO_3^{2-} anions.

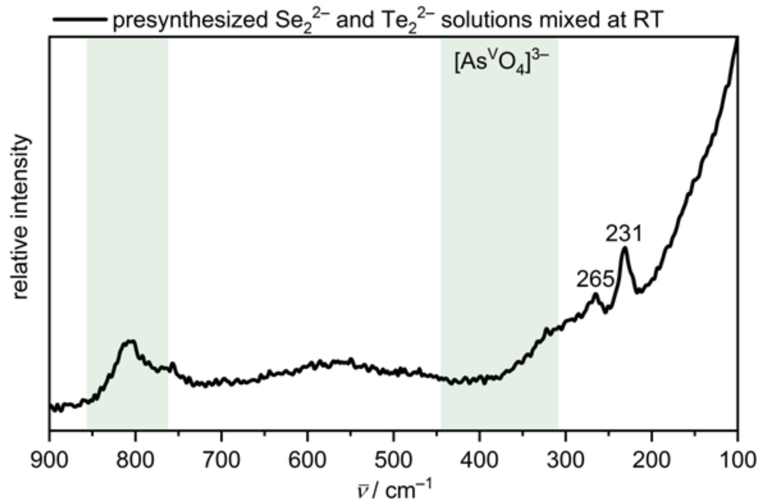

Figure S17. Raman spectra of mixed chalcogenide solutions obtained by mixing a Se_2^{2-} solutions synthesized with q(Se) = 1 and a Te_2^{2-} solutions synthesized with q(Te) = 1 at room temperature. The initial concentrations of these dichalcogenide solutions was equal, however, the crystallization of K_2Te_3 lowered the Te_2^{2-} concentration.

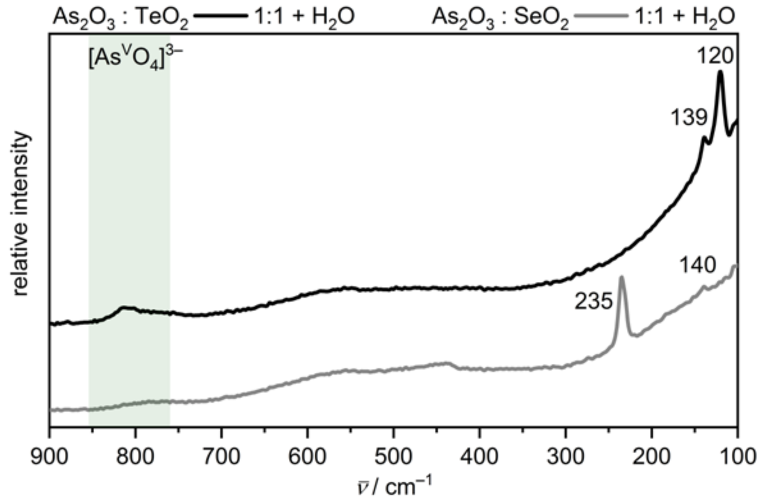

Figure S18. Raman spectra of hydroflux synthesis with *q*(Se) = 1 and *q*(Te) = 1, which were mixed with water and stored at air until the color of the solutions vanished. The telluride solution quickly decomposed to a gray powder, while a red film slowly formed on the surface of the selenide solution, which over the course of hours turned into a gray solid that sank to the bottom of the beaker.

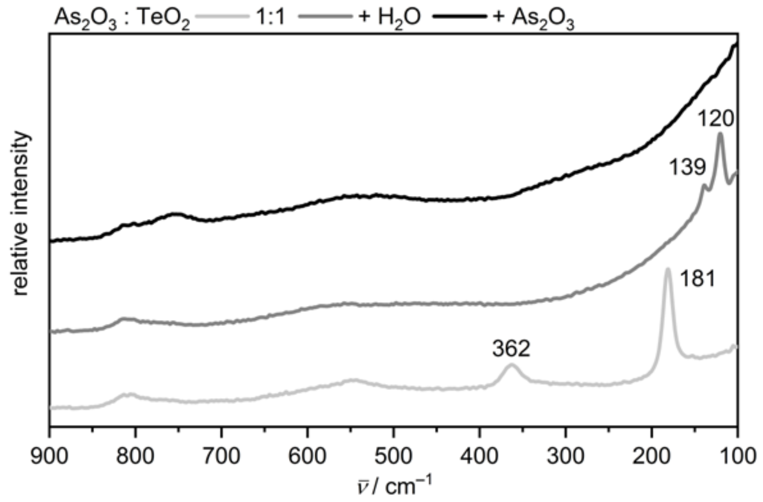

Figure S19. Raman spectra of a Te_2^{2-} solution getting decomposed by water resulting in elemental Te, which is reduced by dissolved As_2O_3. The band at 362·cm^{-1} is the first overtone of the Te_2^{2-} vibration band at 181 cm^{-1}. The bands at 139 cm^{-1} and 120 cm^{-1} are assigned to elemental Te.

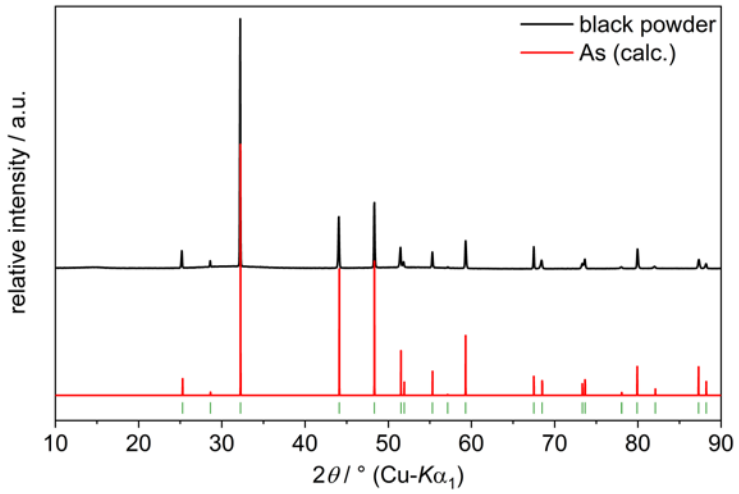

Figure S20. Powder X-ray diffractogram of the black powder found at the bottom of the PTFE inlet of synthesis with $q(Ch)$ of 2 or larger in comparison with a calculated pattern of elemental arsenic (ICSD: 16518).

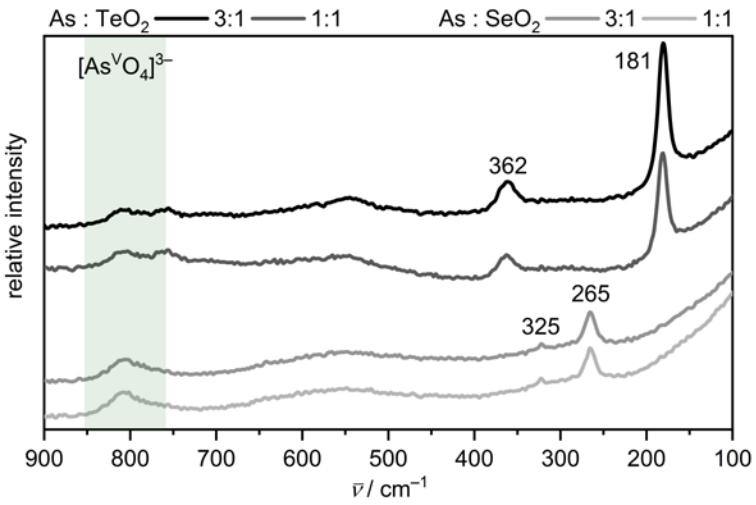

Figure S21. Raman spectra of chalcogenide solutions obtained by hydroflux syntheses with different As:TeO_2 and As:SeO_2 ratios by starting from elemental As instead of As_2O_3. The band at 362 cm^{-1} is the first overtone of the Te_2^{2-} vibration band at 181 cm^{-1}. The Se_2^{2-} band occurs at 265 cm^{-1}, while the 325 cm^{-1} is assigned to the Se_2^{-} radical.

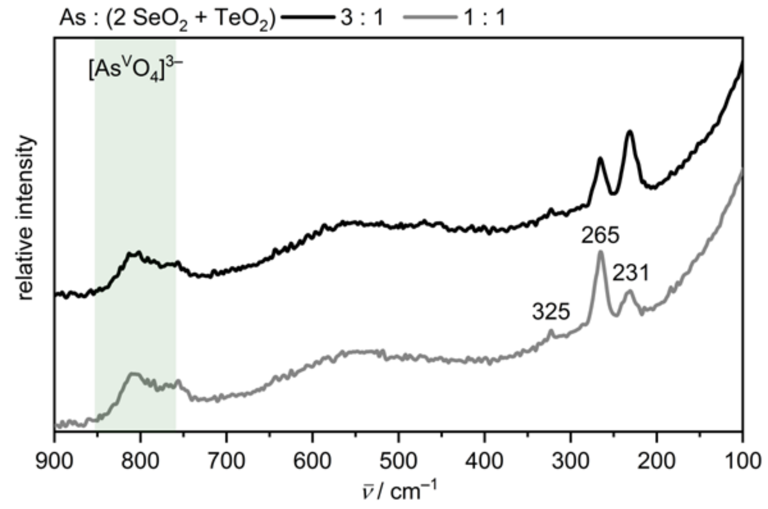

Figure S22. Raman spectra of chalcogenide solutions obtained by hydroflux syntheses with different As:($2SeO_2$+TeO_2) ratios by starting from elemental As instead of As_2O_3. The band at 231 cm^{-1} is caused by the $SeTe^{2-}$ anion. The Se_2^{2-} band occurs at 265 cm^{-1}, while the 325 cm^{-1} is assigned to the Se_2^{-} radical.

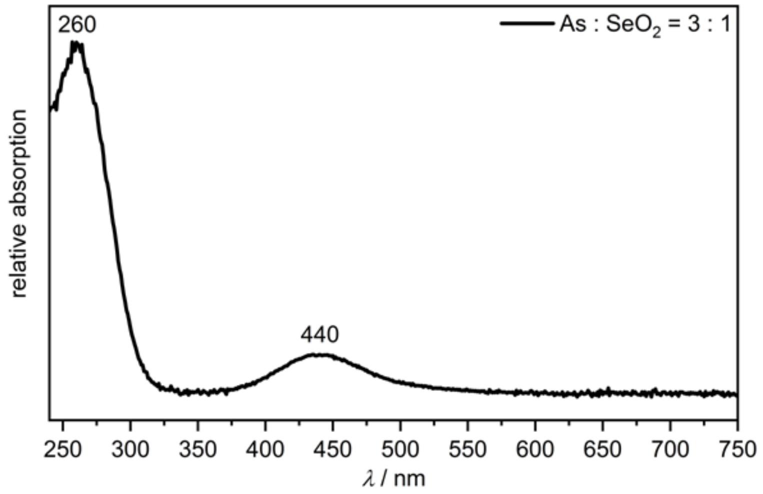

Figure S23. UV-Vis spectrum of orange selenide solution obtained by hydroflux synthesis with a large excess of arsenic as reducing agent for SeO_2 measured in reflection mode. The intensity of the band at 260 nm indicate a low concentration of the monoselenide anions, since the monochalcogenide anions have an absorption about 10 times greater than the dichalcogenide ones.[6]

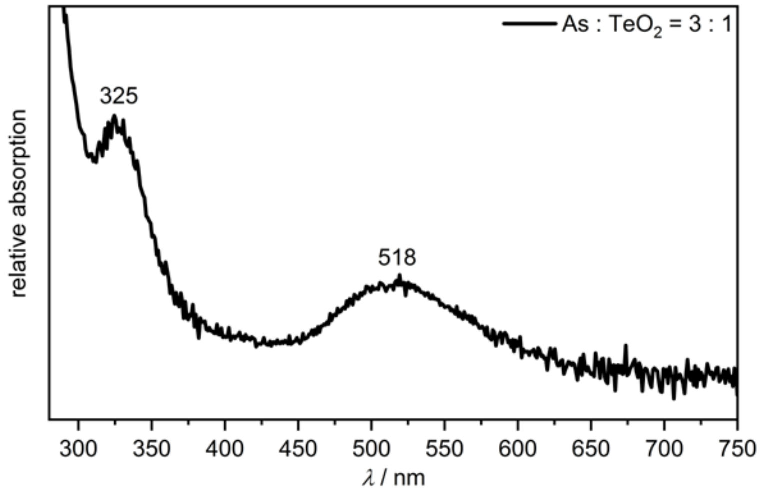

Figure S24. UV-Vis spectrum of purple telluride solution obtained by hydroflux synthesis with a large excess of arsenic as reducing agent for TeO_2 measured in reflection mode. The intensity of the band at 325 nm indicate a low concentration of monotelluride anions, since the monochalcogenide anions have an absorption about 10 times greater than the dichalcogenide ones.[6]

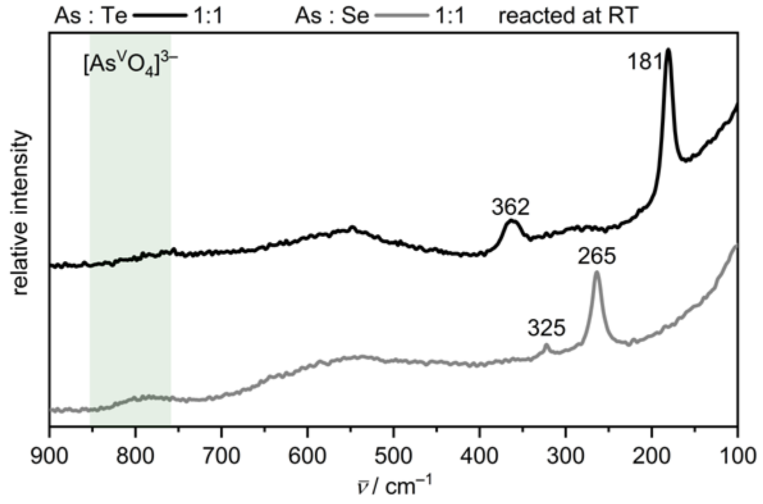

Figure S25. Raman spectra of chalcogenide solutions obtained by hydroflux syntheses with As:Se and As:Te ratios of 1 after adding potassium hydroxide. The band at 362 cm^{-1} is the first overtone of the Te_2^{2-} vibration band at 181 cm^{-1}. The Se_2^{2-} band occurs at 265 cm^{-1}, while the 325 cm^{-1} is assigned to the Se_2^{-} radical.

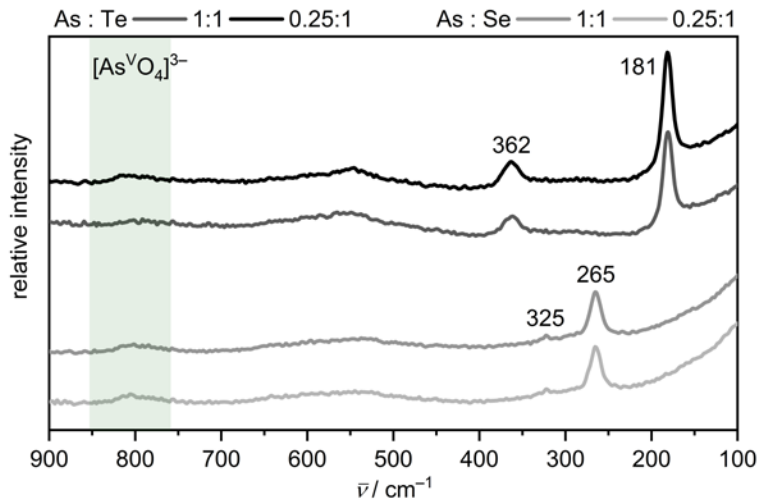

Figure S26. Raman spectra of chalcogenide solutions obtained by hydroflux syntheses with different As:Se and As:Te ratios by starting from the elements instead of the respective oxides. The band at 362 cm^{-1} is the first overtone of the Te_2^{2-} vibration band at 181 cm^{-1}. The Se_2^{2-} band occurs at 265 cm^{-1}, while the 325 cm^{-1} is assigned to the Se_2^{-} radical.

References

[1] *APEX2*, Bruker AXS Inc., Madison, Wisconsin, USA, **2014**.
[2] G. M. Sheldrick, *Sadabs: Area-Detector Absorption Correction*, Bruker AXS Inc., Madison, Wisconsin, USA, **2014**.
[3] G. M. Sheldrick, *Acta Crystallogr., Sect. A* **2015**, *71*, 3–8.
[4] G. M. Sheldrick, *Acta Crystallogr., Sect. A* **2008**, 112–122.
[5] K. Brandenburg, *Diamond 4, Crystal and Molecular Structure Visualization*, Crystal Impact GbR, Bonn, Germany, **2017**.
[6] L. E. Lyons, T. L. Young, *Aust. J. Chem.* **1986**, *39*, 511–527.

Author Contribution

R. A. carried out the syntheses and characterization of the samples, analyzed the data and wrote the original draft of the manuscript. M. R. supervised the project, acquired the funding and wrote the manuscript.
